# Parametric study of pulsed nanosecond laser interaction with carbon-nanotube composite bipolar plate for PEMFCs

**DOI:** 10.1038/s41598-023-28700-2

**Published:** 2023-02-04

**Authors:** Dawit Musse, Dongkyoung Lee

**Affiliations:** 1 Department of Future Convergence Engineering, Cheonan College of Engineering, Kongju National University, Cheonan, 31080 South Korea; 2 Department of Mechanical and Automotive Engineering, Cheonan College of Engineering, Kongju National University, Cheonan, 31080 South Korea; 3 Center for Advanced Powder Materials and Parts of Powder (CAMP2), Cheonan College of Engineering, Kongju National University, Cheonan, 31080 South Korea

**Keywords:** Mechanical engineering, Materials for devices

## Abstract

A laser processing technique is proposed for the processing of a 2.5 mm thick carbon nanotube (CNT) composite bipolar plate for proton exchange membrane fuel cells (PEMFCs). This study aims to understand laser interaction with the CNT composite plate experimentally using a pulsed nanosecond laser. Penetration depth, top width, spatter width, and overall physical morphologies are studied. Scanning electron microscope (SEM) and 3D Scanning Confocal Microscope were used for observation and measurements. Based on that, a parametric investigation is conducted and reported systematically. Most importantly, the pulse repetition rate presents a unique nature of interaction that resulted in a critical repetition rate distinguishing three operational regimes. The physical and chemical properties of the regimes are further analyzed by Vickers microhardness testing and energy dispersive X-ray (EDX) analyses performed on the surface and cross-section of each specimen. The results reveal that the pulse repetition rate introduces changes in mechanical properties and chemical compositions in the vicinity of the processed region. In conclusion, lower pulse repetition should be favored for less impact on mechanical properties, chemical composition, and morphological aspects.

## Introduction

Carbon nanotubes (CNTs) have remarkably outstanding mechanical properties (elastic modulus and tensile strength) and superior electrical and thermal conductivities, making them stiff and strong conducting materials with low weight compared to steel and other structural materials^1^. This stimulated a great deal of attention in the advanced composite society in their use as reinforcement materials for the advancement of composite materials^2^. These composites are being used for various applications in wearable systems (smart textiles), robotics, and next-generation electronics devices and energy conversion systems^3–5^. In addition to the remarkable mechanical, electrical, and thermal properties, CNTs possess high surface-to-volume ratios resulting from their small diameters, which are order of few nanometers. This has created a great opportunity for CNT composites, particularly in battery and energy conversion devices where increased effective contact surface area of electrodes per volume plays a significant role in energy conversion efficiency^6–8^. CNTs are identified as potentially useful enforcement in lithium-ion battery systems, fuel cells, and solar cells^9–11^. A CNT composite with a metal nanoparticle as an electrode doubles the performance of hydrogen fuel cells due to the increased catalytic activity of carbon nanotube-based electrodes^12,13^. Other studies pointed out the relevance and applications of CNTs in lithium-ion batteries^14^, elastic and transparent conductive films^15^, and flat panel displays^16^.

Following the increasing demand and applicability of CNTs in various technological fields, developing effective manufacturing processes is vital for processing CNT composites to the desired size, shape, and quality. Any manufacturing method selected for processing CNT composites must ensure minimum damage to the CNT structure that could result from pressure, heat, or chemical reaction with the matrix material. Conventional processes like machining and molding are associated with certain disadvantages. Since CNTs have high strength and hardness, conventional machining methods would cause intensive tool wear, reducing the tool life and increasing production cost^17^. The alignment of CNTs in molded composites is significantly affected by the shear flow in the molding process leading to undesired changes in its structures and properties^18^.

The continuous advancements in the performance of lasers during the past decades have improved their capacity in various fields, including energy, biotechnology, electronics, and mechanical engineering^19^. In polymeric composite cutting, lasers offer variety of advantages, including high production speeds without shortcomings associated with tool wear and vibration^20,21^. Lasers are specifically advantageous in the processing of difficult-to-machine materials^22^, like carbon fiber composite and graphite composites due to their brittleness and hardness.

Many researchers have studied the use of lasers in processing CNTs and their composites. Wu et al.^23^ performed the ablation and patterning of CNT film using a femtosecond laser for applications in electroluminescent and flexible electronic components. Raman spectroscopy and scanning electron microscope (SEM) were used to characterize the performance of the pattern groove. The research indicated the influence of process parameters on ablation and pattering at different pulse energies. In addition, appropriate cutting parameters that introduce minimum defects were suggested. Chen et al.^24^ studied the structural modification and transformation of CNT by using ND: YAG laser operating at 266 and 1067 nm wavelengths, with different energy fluences and the number of passes to study the structural modification of CNT. Accordingly, it was demonstrated that transformation on a selective area could be effectively controlled by laser fluence and the number of passes. Effects of processing parameters on laser cutting of multi-walled carbon nanotubes/polymethyl methacrylate nanocomposites are investigated by Azmats et al.^25^ for applications of CNTs as a reinforcement for plastics to take advantage of their good mechanical, geometrical, electronic, and electromagnetic properties. Furthermore, the research suggested that the number of CNT is an influential factor in reducing HAZ by up to 50%. Overall, these researches emphasized the need of packing of CNTs into microscopic materials: films, sheets, and ribbons that are flexible and thin structures and need to be cut or structured into nanometer and micrometer dimensions using lasers. On the other hand, CNTs are also packed into materials at the macro level to produce CNT composites for applications such as bipolar plates of PEMFCs for their effective charge transfer and thermal management capabilities^26^. Developing effective manufacturing methods for bipolar plate is very crucial because bipolar plate is a key component in fuel cells with multiple functional roles, contributing up to 40–45% of the total fuel cell cost^27,28^. The current technological advancement has introduced a variety of advanced lasers with a variety of capacities and applications that can remedy the challenges in the machinability of CNT composite bipolar plates due to their brittleness and hardness. The use of direct laser melting (DLM) technology to fabricate the flow channels/patterns of metallic bipolar plates of direct methanol fuel cell (DMFC) was identified from the work of Moon et al.^29^ who compared its performance to numerical control (NC) machined bipolar plate. Results show that the overall fuel cell performance increases for DLM micro-patterned bipolar plate because of rough side walls from the laser operation that causes more rapid chemical reactions. Despite these earnest efforts and achievements, no attempt has been made to overcome the challenge of flow channel fabrication on CNT composite bipolar plates using lasers.

In this study, laser processing is proposed for the manufacturing of fuel and oxidizer flow channels on a CNT composite bipolar plate made by blending graphite, epoxy, and CNTs. However, understanding the laser-material interaction is an essential step and should be a prerequisite condition before proceeding with the suggested solution. Therefore, this paper aims to clearly understand the interaction of nanosecond laser with CNT composite bipolar plate. To assess the performance of laser in the processing of CNT composites, the effects of power, scanning speed, the number of passes, repetition rate, and pulse duration on the penetration depth, top width, spatter width (if spatter forms) and physical morphology of the specimen are systematically analyzed and discussed. Furthermore, chemical characterizations and mechanical property measurements are performed for parametric settings that yield special morphological appearances.

## Materials and methods

**Figure 1:**
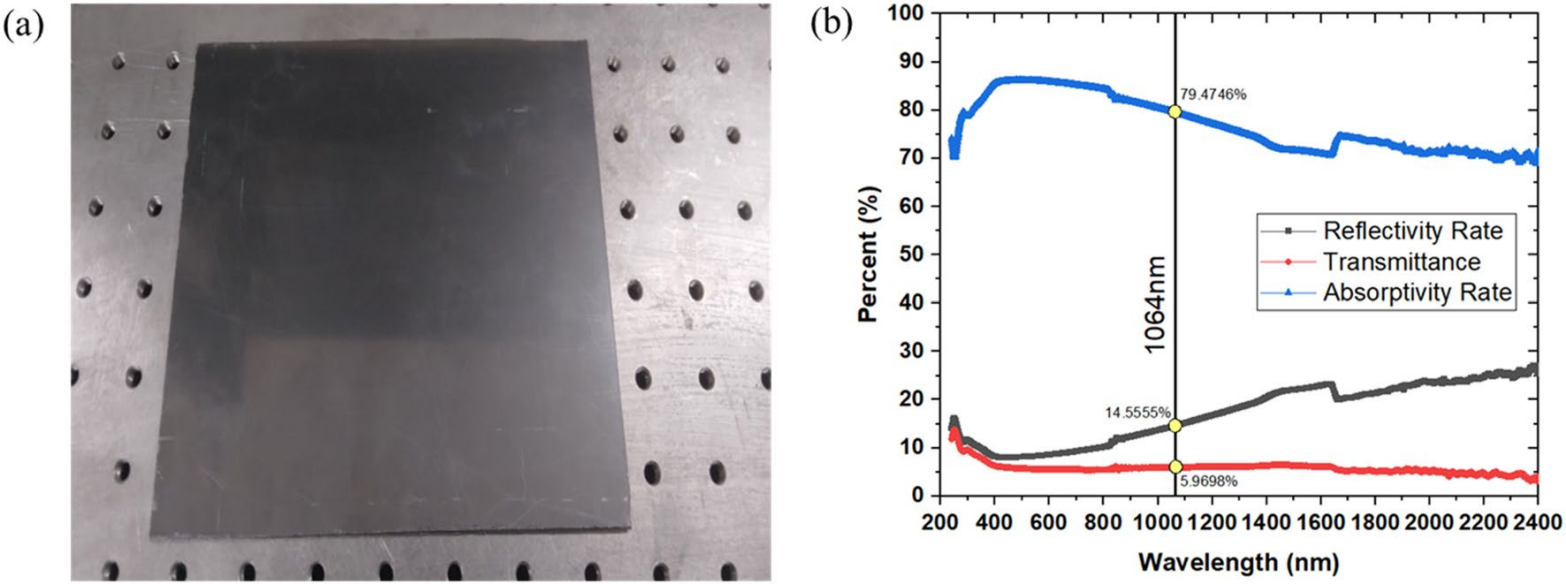
(**a**) A CNT composite plate (**b**) reflectivity, absorptivity and transmittance of the plate in (**a**).

**Figure 2:**
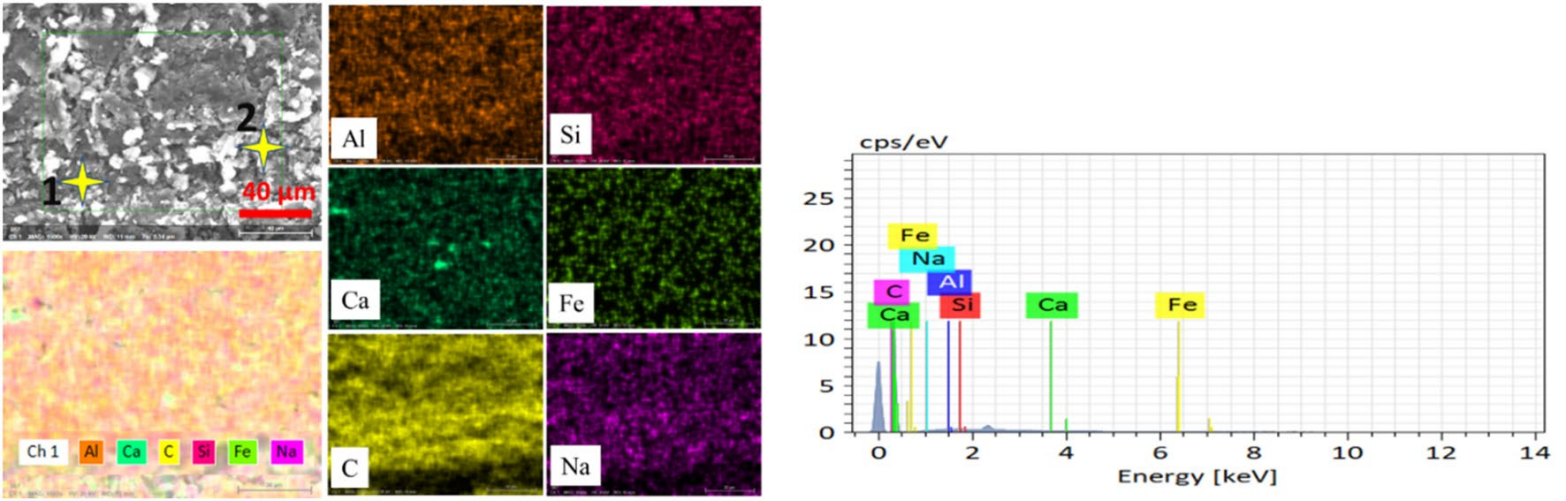
EDX mapping of unprocessed base material.

Samples are prepared from a 2.5 mm thick CNT composite plate that is manufactured for bipolar plates in PEMFCs, shown in Fig. 1a. The CNT composite plate material is fabricated from graphite (77% by weight), a polymer, specifically, a thermoset (epoxy) polymer (20% by weight), and CNTs as filler materials (3% by weight). The CNTs are highly conductive multi-walled CNTs with a diameter of 11–13 nm, a length of 40–50 μm, a purity of 95 wt.%, and a bulk density of 0.025 g/ml. A molding process at 50 MPa molding pressure was employed during its production. Optical properties of the material are of immense importance for its laser processibility. Figure 1b presents the UV–Vis-NIR spectrophotometer analysis of the sample for the reflectivity rate, absorptivity rate and transmittance of the 2.5 mm thick CNT composite plate sample at various wavelengths. A chemical characterization of the plate (which can be referred as unprocessed base material) is done using EDS analysis, and elemental mappings are shown in Fig. 2. The average values of elemental compositions are tabulated in Table 1.

**Table 1:** Elemental composition of the base material.

Element	Composition (Weight %)
C	82.67
Al	7.475
Si	4.145
Fe	2.9
Miscellaneous (Nama)	2.805

### Experimentation

**Figure 3:**
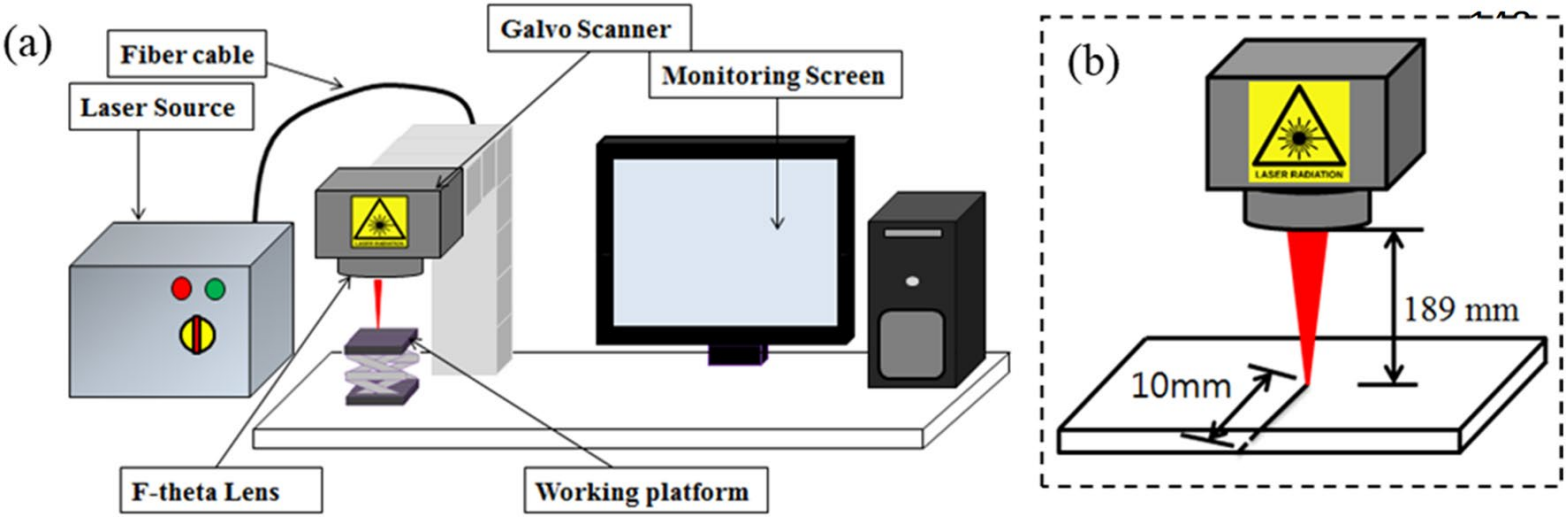
(**a**) Experimental setup and (**b**) laser irradiation path.

Ytterbium nanosecond pulsed Nd:YAG fiber laser (IPG-YLPM, IPG photonics, model YLP-HP IPG photonic, Southbridge, MA, USA) was used as a laser source. The laser source has an emission wavelength of 1064 nm, generating up to 20W average power, 1000 kHz repetition rate, 200 ns pulse duration, and a scanning speed of 2000 mm/s. The source has a beam quality factor (M^2^) of 1.5, a collimated beam diameter of 12.8 mm and a spot diameter of 30 μm at 189 mm focal distance. Figure 3a illustrates the schematics of the experimental setup. Preliminary experiments suggested that ablation of CNT composites was achieved from low laser powers to the highest [4–20 W] and from low scanning speed to moderate values [up to 300 mm/s]. For this reason, laser powers of 4–20 W with an interval of 2W and scanning speeds of 50–300 mm/s at an interval of 50 mm/s were considered. Each pulse duration (4, 20, 50, 100, and 200 ns) has its own set of working frequency ranges on the pulsed laser. For instance, with lower pulse duration values, the operation can only be performed at higher repetition rate values, and operations on lower repetition values became possible at higher pulse duration settings. Hence, at 200 ns, all frequency settings [20–1000 kHz] are active. So, typical frequency values for the onset of new pulse duration (20, 40, 60, 105, 500, and 1000 kHz) were considered for the study. The experimental parameters are presented in Table 2. The same table can be referred to understand frequency and pulse duration couplings. To understand the interaction between the laser and the CNT composite plate, laser irradiations are performed by line exposures on the specimen in a configuration given in Fig. 3b. Line irradiations are simple and proper ways to examine the ablation characteristics and understand the laser interaction with the material.

**Table 2:** Experimental parameters.

Parameters	Fixed values	Peak pulse intensity (kW/mm^2^)
Power [W]: 4–20W (Interval: 2)	200 ns, 100 mm/s, 20 kHz, 1 pass	1414.93–7074.64
Scanning speed [mm/s]: 50–300 mm/s (Interval: 50)	20W, 200 ns, 20 kHz, 1 pass	7074.64 (constant)
Number of passes [-]: 1–20 Passes (Interval: 4)	20W, 200 ns, 100 mm/s, 20 kHz	7074.64–141,492.80
Repetition rate [kHz] and pulse duration [ns]		
20, 40, 60, 105, 500, 1000	20W, 100 mm/s, 1 pass, 200 ns	7073.55–141.471
40, 60, 105, 500, 1000	20W, 100 mm/s, 1 pass, 100 ns	7073.55–282.94
60, 105, 500, 1000	20W, 100 mm/s, 1 pass, 50 ns	9431.67–565.90
105, 500, 1000	20W, 100 mm/s, 1 pass, 20 ns	13,473.434–1417.71
500, 1000	20W, 100 mm/s, 1 pass, 4 ns	14,147.11–7073.5

### Measurements

**Figure 4:**
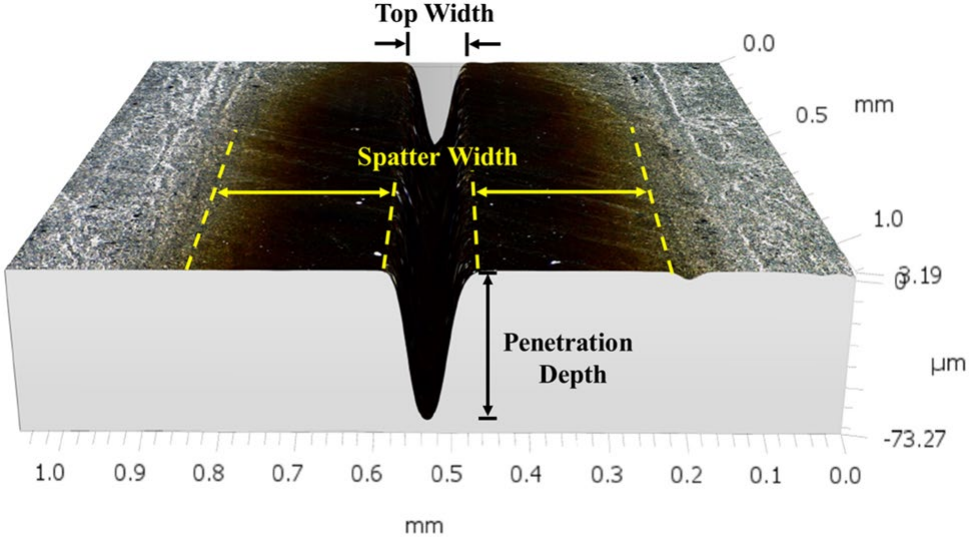
Illustrations of top width, penetration depth and spatter width.

**Figure 5:**
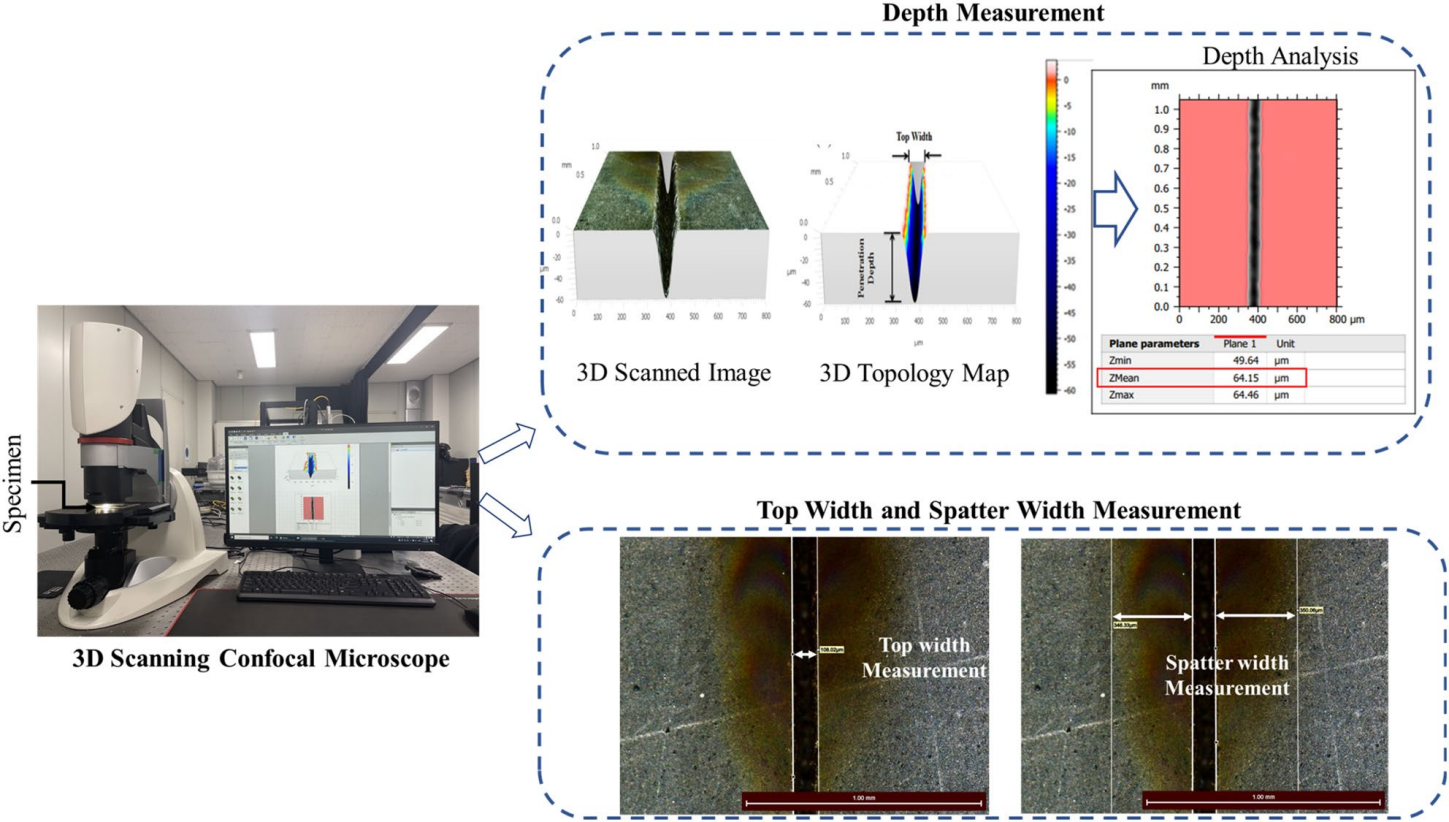
Measuring device and measurement methods.

The response parameters are penetration depth, top width, and spatter width. The penetration depth is the actual depth created by the laser beam, while the top width is the maximum width of the material removed by the laser. Spatter width is defined as the average width of the spatter dispersed in the vicinity of the processed region. Measurement results are obtained from a high-precision 3D scanning Leica Confocal Microscope (Leica PLANAPO FOV 3.6, DMI, USA). The digital microscope has magnification from 12x to 2340x depending on the objective selected (low or high). 2340x (high magnification) can show details down to 0.4 µm. It has 10 megapixel integrated high resolution camera with integrated ring ling and coaxial LED illumination and a tilting stand (− 60° to + 60°). 3D topology map from the confocal digital microscope can capture topological variations at inner section of the groove and also it captures possibilities of ablated particles trapping and molten spatter formation inside the groove. The 3D scanner can analyze a large area and calculate the mean depth, which provides a more reliable data there by avoiding the need for repeated experiments. Accordingly, the study is conducted on a full factorial experimental design based on an experiment performed on each parametric combination. The response parameters and the measurement device with the measurement methods are schematically illustrated in Figs. 4 and 5, respectively. In addition, SEM was used to thoroughly observe and report important morphological feature resulting from the interaction with the laser. In cases when chemical and mechanical property characterizations were important, Electron-dispersive X-ray Spectroscopy (EDX) and Vickers Microhardness Tester are employed. EDX analysis is also carried out for the characterization of the spatter with respect to the base material. The apparatus used for SEM and EDS is MIRA 3-LMH High Resolution Emission Scanning Microscope with high brightness Schottky emitter electron gun (manufactured by Tescan). It has a resolution of 1.0 nm at 30 kV and 2.0 nm at 3 kV and a magnification up to 1,000,000 × and magnification up to 4 × without any image distortion in wide field optic mode. Additional specifications include, accelerating voltage of 200–30 keV and probe current of 1 pA to 100 nA. The Vickers microhardness tests are conducted with a microhardness testing machine (HM, Mitutoyo Corporation, Japan) to determine the hardness or specimen’s resistance to deformation. The CNT composite plate manufactured with a very smooth finish (measured roughness of 0.1534 μm), therefore, it does not require polishing. The Vickers hardness tester uses a diamond indenter that applies a 0.49N force (50 g-force) on the surface of the specimen for a dwell time of 10 s.

## Results and discussion

### Effect of power and scanning speed

**Figure 6:**
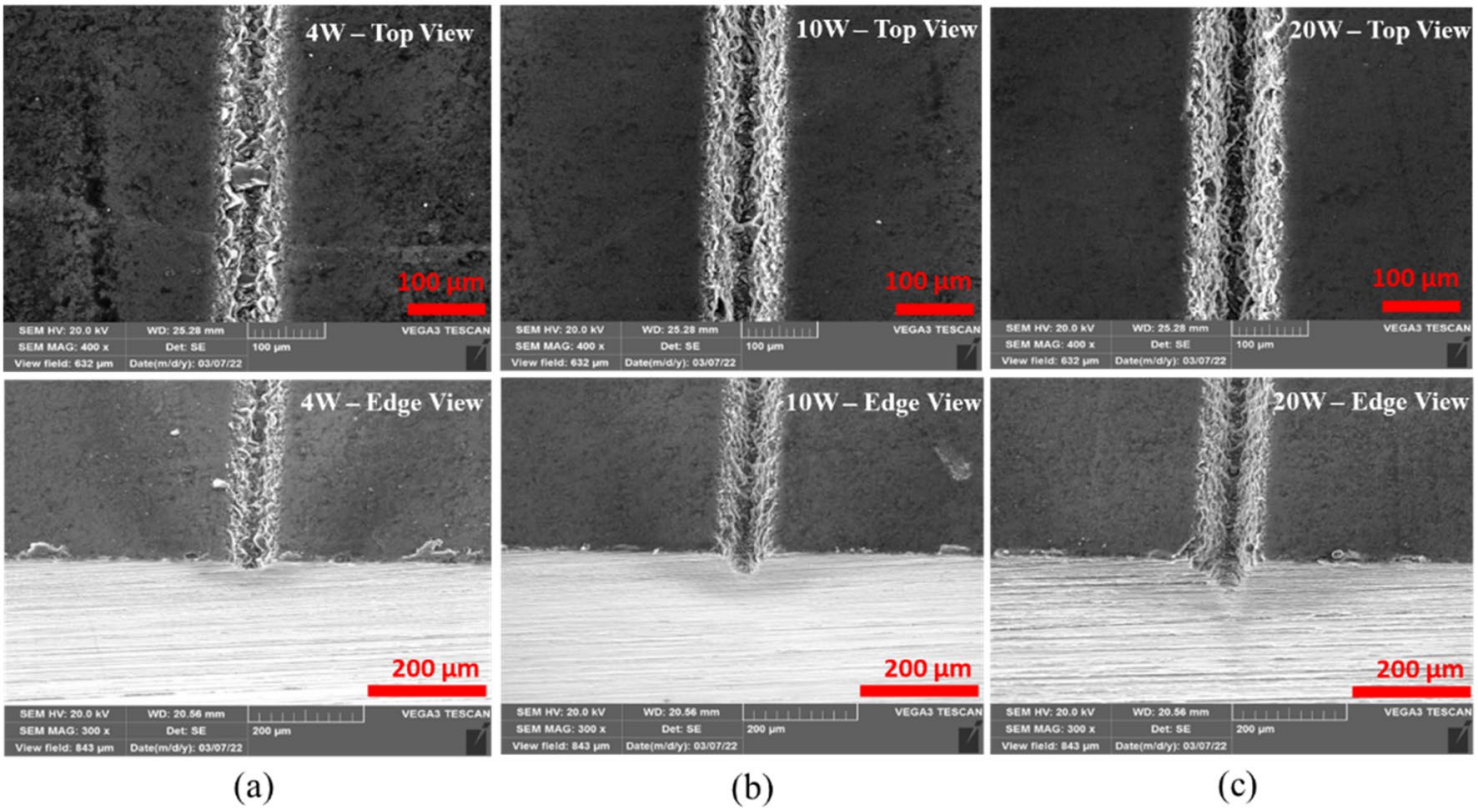
Effect of power on physical morphology; (**a**) 4W, (**b**) 10W, (**c**) 20W [Fixed: 20 k Hz, 200 ns, 100 mm/s and 1pass].

**Figure 7:**
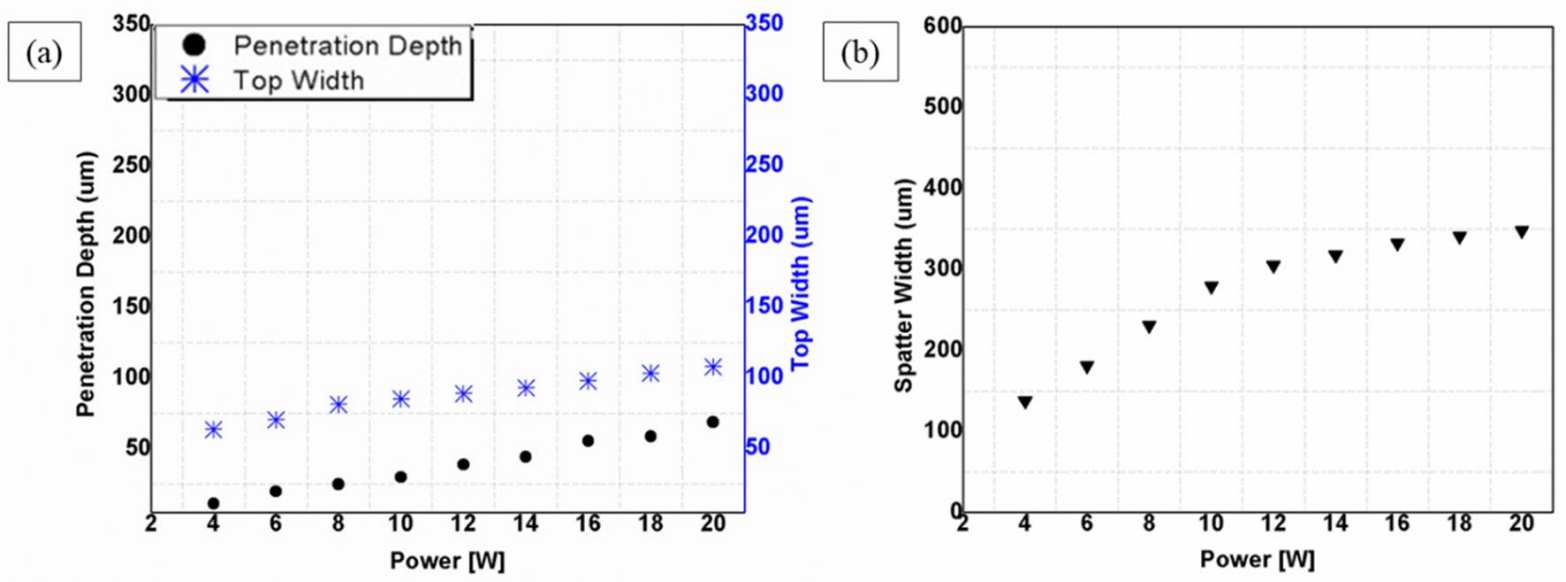
Effects of power; (**a**) Effect on top width and penetration depth, (**b**) Effect on spatter width [Fixed: 20 kHz, 200 ns, 100 mm/s and 1pass].

Laser power (average laser power) is a measure for the energy of light delivered by the beam per unit time, which is a commonly used parameter to control a laser process^30^. This section presents the effect of laser power on laser processing of CNT composites from 4 to 20W at fixed parameters of 200 ns, 20 kHz, 100 mm/s and 1 pass and the effect of scanning speed from 50 to 300 mm/s at fixed parameters of 20W, 200 ns, 20 kHz and 1 pass. In laser processing of CNT composite, it was observed that low to high powers could be used depending on the extent to which the material is needed to be ablated. Experimental results show that power causes both penetration depth and top width to increase. Figure 7a illustrates the effect of power on the penetration depth and top width. Furthermore, the effect of power on the physical morphology of the sample was observed. Based on that, no significant effects were observed. Physical morphologies of the specimens at low power (4W), medium power (10W) and high power (20W) are given in Fig. 6a, b and c.

It was identified that most parametric combinations in laser processing of CNT composites lead to the dispersion of spatter from the processed zone to the periphery of the laser irradiated line. Observations made on the specimens before and after the cleaning of the spatter is presented in Table 3. Cleaning of the spatter is done by careful wiping using a general cleaning wet microfiber cloth to avoid skin contact and inhaling by the worker and for the safety of the specimen as well. Specific issues such as causes of spatter formation and characterization of the spatter are discussed in the upcoming sections. According to the study conducted on the formation of spatter (in "Effect of pulse duration and repetition rate" Section), it was indicated that repetition rate is the key parameter for spatter formation. More specifically, lower repetition rates produce a spatter of larger width. Since the effect of power is studied at 20 kHz, which is a pulse repetition rate that gives off abundant spatter, it was possible to observe spatter. The relationship between power and the resulting spatter width can be seen in Fig. 7b. It could also be seen that more spatter is formed due to the increase in material removal as power increases.

**Table 3:** Relationship of the spatter with power.

Power	4W	10W	20W
Image description			
Spatter zone			
Wiped

**Figure 8:**
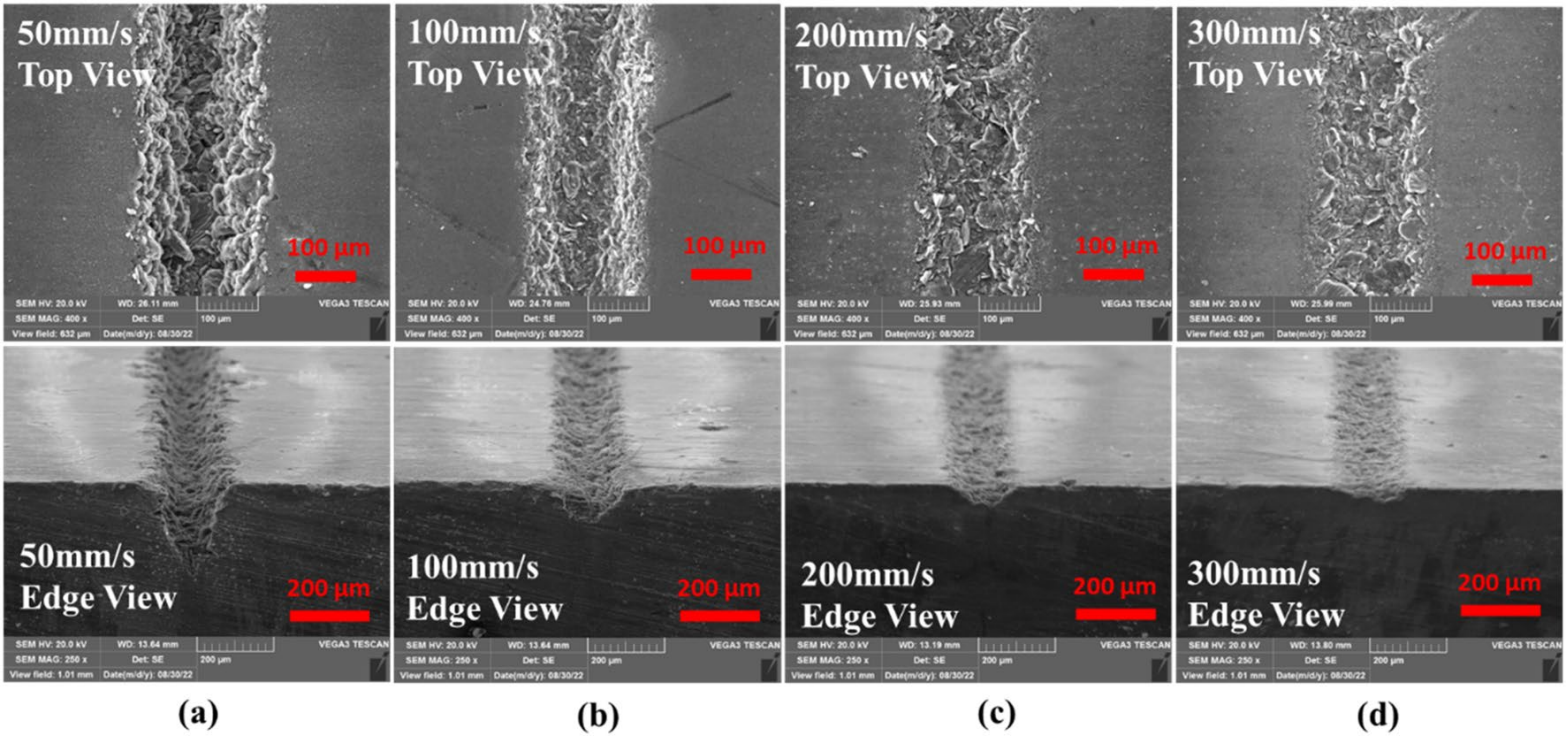
Effect of scanning speed on physical morphology; (a) 50 mm/s, (b) 100 mm/s, (C) 200 mm/s (d) 300 mm/s [Fixed: 20 kHz, 200 ns, 20W and 1pass].

**Figure 9:**
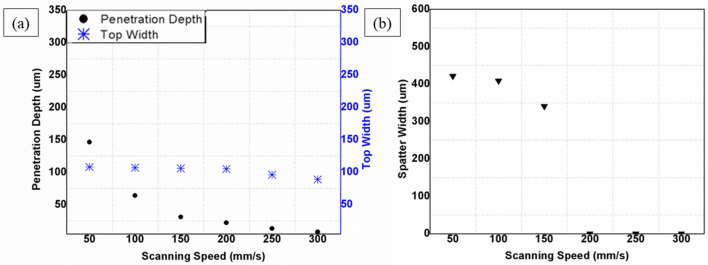
Effects of scanning speed; (**a**) Effect on top width and penetration depth, (**b**) Effect on spatter width [Fixed: 20 kHz, 200 ns, 20W and 1pass].

Scanning speed represents the duration of laser interaction, implying more interaction at low speeds and less at high speeds. In the laser processing of CNT composites, the extended period of interaction at low speeds [50–100 mm/s] causes deeper penetration, but the high-speed operations [150–300 mm/s] cause a shallow penetration. From Fig. 9a, the effect of scanning speed is observed to be primarily on the penetration depth, and its effect on top width is almost negligible. Scanning speed presents no significant morphological changes on the CNT composite. As shown in Fig. 8a, low scanning speed gives a longer interaction that offers high penetration depth. Increasing the scanning speed to 100, 200, and 300 mm/s, as in of Fig. 8b, c and d respectively, the penetration depth decreases proportionally due to a reduction in ablation that resulted from short interactions with increased scanning speed. In addition, the process is partially spatter-forming (shown in Fig. 9b) which gives off the spatter at low to medium scanning speed levels (50–150 mm/s) only. In contrast, higher scanning speeds (200–300 mm/s) do not result in a significant amount of spatter and in a consistent and well-established form (in a fashion presented in Table 3).

Generally, laser power and scanning speed can be used to control the penetration depth and top width of the laser-irradiated zone in smooth operation without having any form of significant changes in the physical morphology.

### Effect of number of passes

**Figure 10:**
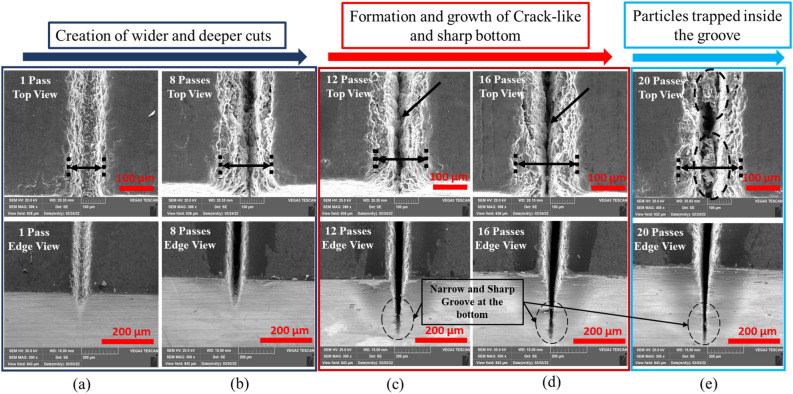
Effect of number of passes; (**a**) 1pass, (**b**) 8passes, (**c**) 12passes, (**d**) 16passes and (**e**) 20passes [Fixed: 20 kHz, 200 ns, 20W and 100 mm/s].

**Figure 11:**
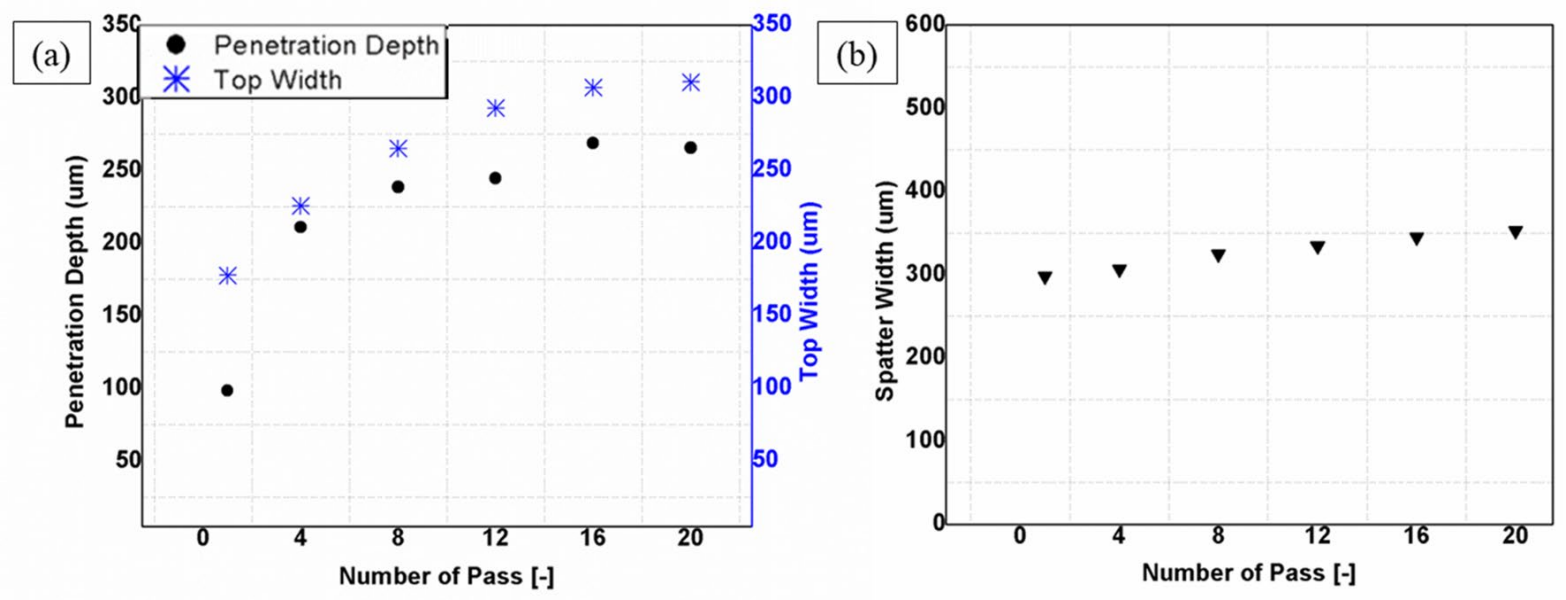
Effect of number of passes at fixed values of 20 kHz, 20W and 20 mm/s; (**a**) Effect on top width (**b**) Effect on spatter width [Fixed: 20 kHz, 20W, 200 ns and 100 mm/s].

**Figure 12:**
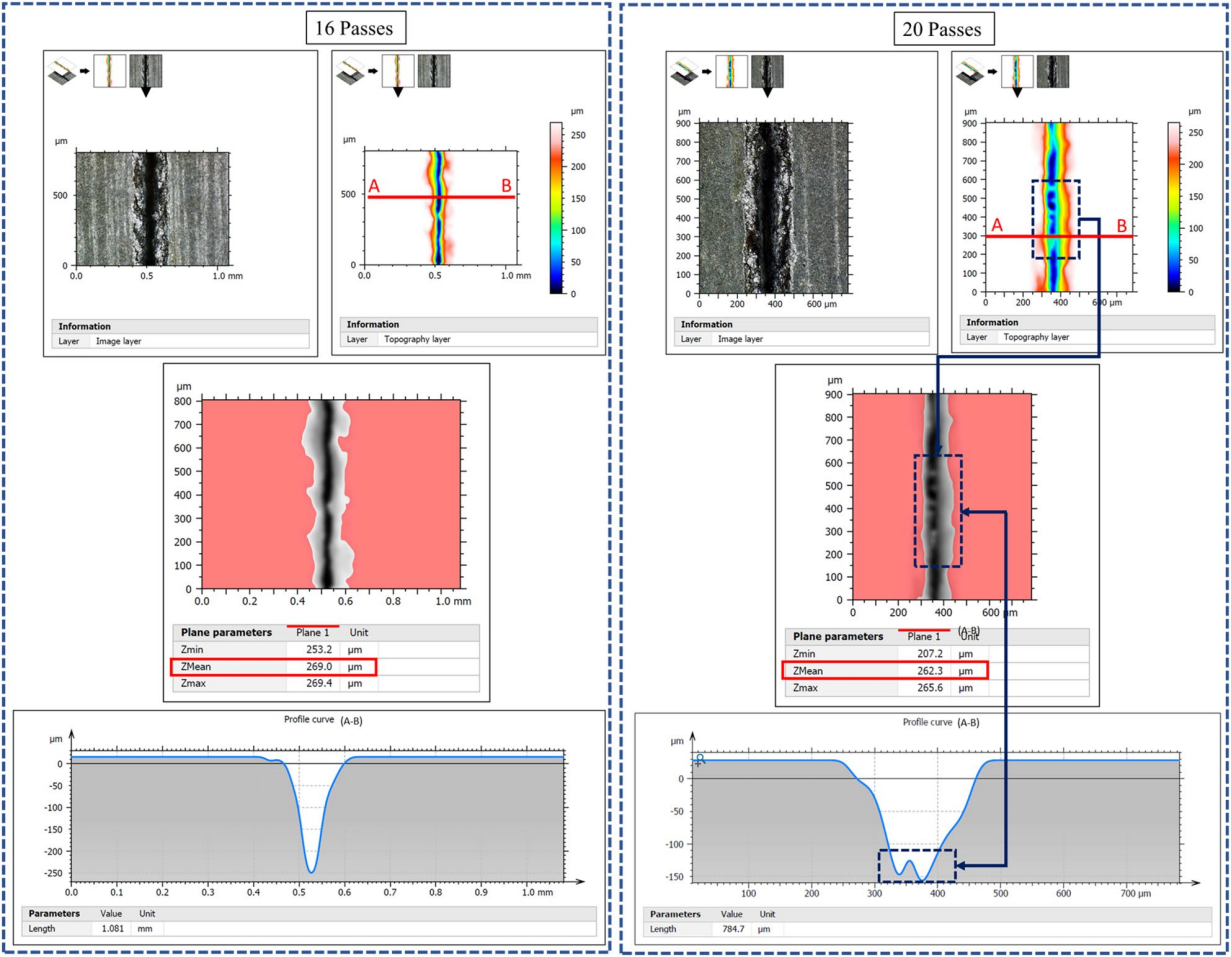
Line profile views and groove morphologies at 16 passes and 20 passes.

The number of passes is a parameter that measures the number of laser irradiation passes. In this section, the effect of number of scan passes is investigated from 1 to 20 passes at 4 passes interval and at a fixed parameters of 20W, 20 kHz, 200 ns and 100 mm/s. In processing CNT composite, the number of passes strongly impacts the processability. As shown in Fig. 11a, a step change in the number of passes greatly affects penetration depth and top width. The formation of wider spatter zones is an additional characteristic feature of the process (Fig. 11b). Additionally, each number of pass display unique forms of a particular morphology. Three main phenomena are observed here. First, at a lower number of passes (up to 8 passes), the ablation becomes powerful to produce deep and wide grooves, as shown in (a) and (b) of Fig. 10. Here, the heat from the laser beam diffuses significantly in the lateral (horizontal) direction and causes material removal from the edges to yield wider channels near the top. Gradual increase of the number of passes to higher values (12 passes), as in the edge view of the case (c) of Fig. 10, begins to initiate crack-like narrow grooves (sharp groove) at the bottom. This effect increases with the number of passes to 16, as in case (d) of Fig. 10, where the effect is amplified with the creation of a wider grooves at the top and sharp/pointy at the bottom. This condition cannot last longer because increasing the top width with the creation of an even narrower groove leads to the deposition of particles to the inside, as shown in case (e) of Fig. 10. These trapped particles could originate from two mechanisms: (1) due to the increase in top width leading to the formation of the large number of particles ablated from the top surface that scatter into the narrow and sharp groove, and (2) due to very high number of the pass at scanning speed of 100 mm/s, that gives less time for removal of particles from the narrow groove (Fig. 11). At this point, it will become a little difficult for the laser beam to penetrate more profoundly because of the blockage of pulses by these particles. As a result, the penetration depth is negatively affected after 16 passes. For clarity, 3D topological details and line profiles at 16 passes and 20 passes are given in Fig. 12.

### Effect of pulse duration and repetition rate

**Figure 13:**
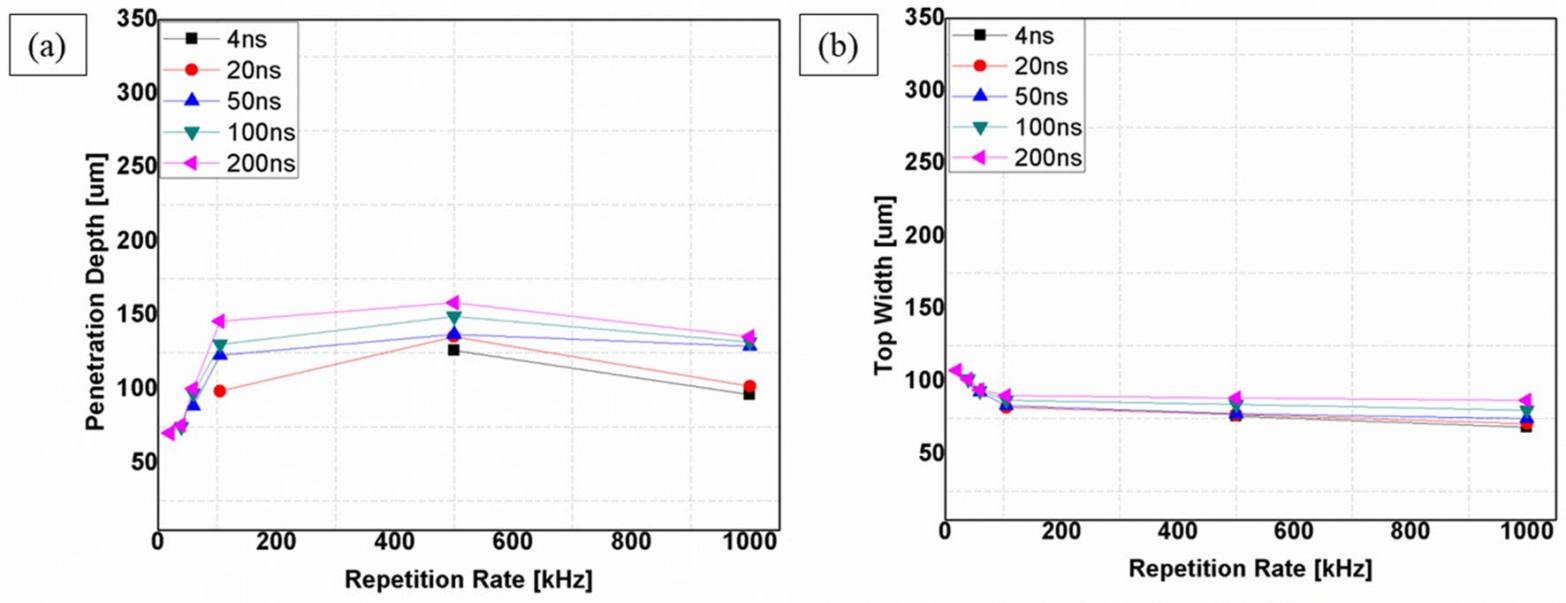
Effect of repetition rate and pulse duration; (**a**) on penetration depth, (**b**) on top width, [Fixed: 20W, 100 mm/s, 1 pass].

**Figure 14:**
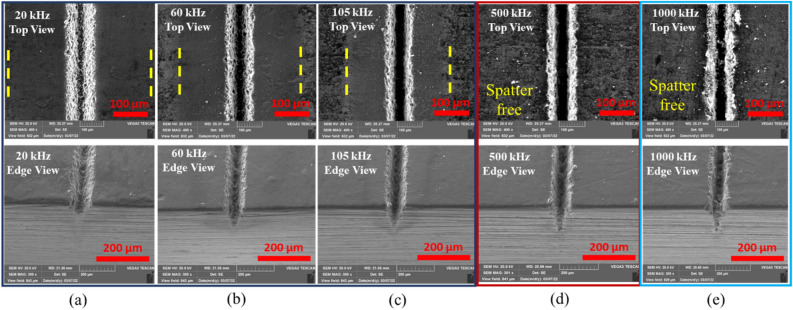
SEM images showing physical morphology with repetition rate at 200 ns. (**a**) 20 kHz, (**b**) 60 kHz, (**c**) 105 kHz, (**d**) 500 kHz and (**e**) 1000 kHz [Fixed: 20W, 100 mm/s, 1 pass and 200 ns].

Pulse duration refers to the time duration between the instant a pulse starts and the instant the pulse ends^31^. This parameter is crucial for pulsed lasers because the laser does work only during this period. The amount of energy applied during this period determines the extent to which the process removes material. Depending on the pulse durations, millisecond, nanosecond, and femtosecond lasers allow flexible adaption of laser processes to treated materials. Within ultrashort pluses, the time to heat the material is less than long pulse lasers or continuous lasers. Therefore, this process will result in the highest precision and lowest damage. This way, ultrashort pulsed lasers enable effective ablation through a cold process^32,33^. However, excessive (long) pulse durations cause more heat to be accumulated at the workpiece, resulting in a thermal process with a significantly larger heat-affected zone^31,34^. Pulse duration is strongly related to pulse repetition rate. In this study, the effects of pulse duration are investigated from 4 to 200 ns at 20W, 100 mm/s, 1 pass and according to pulse duration-frequency coupling of the laser source presented in Table 2, whereas, the effects of pulse repetition rate are studied at 20W, 100 mm/s, 1pass and 200 ns. In the laser processing of CNT composite, pulse duration has imposed an effect on the penetration depth (Fig. 13a). Still, the range of pulse durations used in the experiments (4–200 ns) imposes very little change on the top width; as seen in Fig. 13b. Figure 14 presents the effect of the repetition rate at 200 ns. In Fig. 14a–c, an increase in penetration depth is observed. Additionally, spatter formation with decreasing trend in its width characterizes the operation. This phenomenon continues until 500 kHz, where it attains the maximum penetration depth in a spatter-free process (Fig. 14d). However, further increasing of repetition rate to 1000 kHz (Fig. 14e) will eventually yield a complete change in the morphology and a drop in the penetration depth. The relationship between penetration depth and repetition rate at 200 ns can be referred from Fig. 13a.

**Figure 15:**
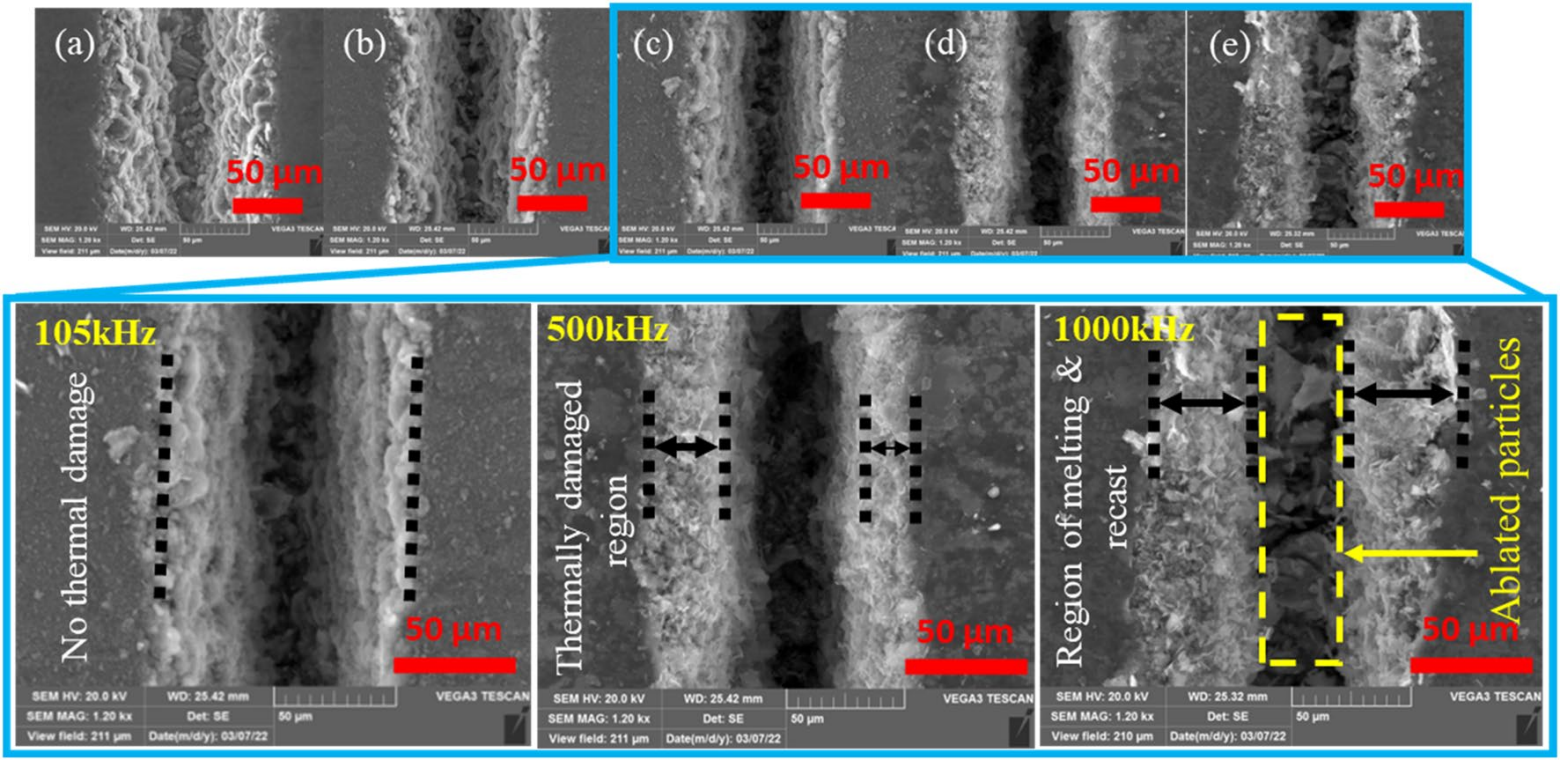
Detailed views of the morphology with repetition rate at 200 ns. (**a**) 20 kHz, (**b**) 60 kHz, (**c**) 105 kHz, (**d**) 500 kHz and (**e**) 1000 kHz, [Fixed: 20W, 100 mm/s, 1 pass and 200 ns].

A close observation of the morphology of five samples in Fig. 14 reveals typical features of the operation. Accordingly, three distinct operational regimes are identified. Regime one consists of cases (a)–(c), 20, 60, and 105 kHz, respectively, which depict operations before the repetition rate of 500 kHz, where a maximum penetration depth is achieved. Operational regime two is identified in case (d) at the 500 kHz. The third operational regime covers the range of repetition rate values above 500 kHz. These categories are well presented in Fig. 15. In regime one, the penetration depth increases with increasing repetition rate. However, the thermal behavior of the process seems to have caused no deformations and as can be confirmed from the physical morphologies of Fig. 15a, b and c. Furthermore, increasing the pulse repetition rate to 500 kHz has resulted in an additional increase in penetration depth (Fig. 13a). Still, close observation of the physical morphology of the sample from Fig. 15d shows that the edges of the groove appear to be thermally damaged. Gradually, unique and interesting characteristics come to an effect as pulse repetition rates are increased beyond 500 kHz. This gives rise to the third operational regime, where the penetration depth drops with an increase in pulse repetition rate. The thermal effect observed for the first time at 500 kHz is advanced to the third regime with more additional effects, as can be observed from Fig. 15e at 1000 kHz. Here, surface layers of the material adjacent to the processed zone are melted, and a recast of these melted layers is formed on its periphery. As discussed above, these three identified regimes have completely unique and different responses to pulse repetition rates causing different ablation characteristics, a gradual increase and decline of penetration depth and morphological features (discovery of thermally damaged and melted regions in regimes two and three). Therefore, it is crucial to conduct further analysis on these three scenarios for a better understanding of the interaction. For this reason, cases of 105, 500, and 1000 kHz are considered representative samples for detailed inspection of the effects on the material's workability.

(i) Operational regime 1—Operation below critical pulse repetition rate

**Figure 16:**
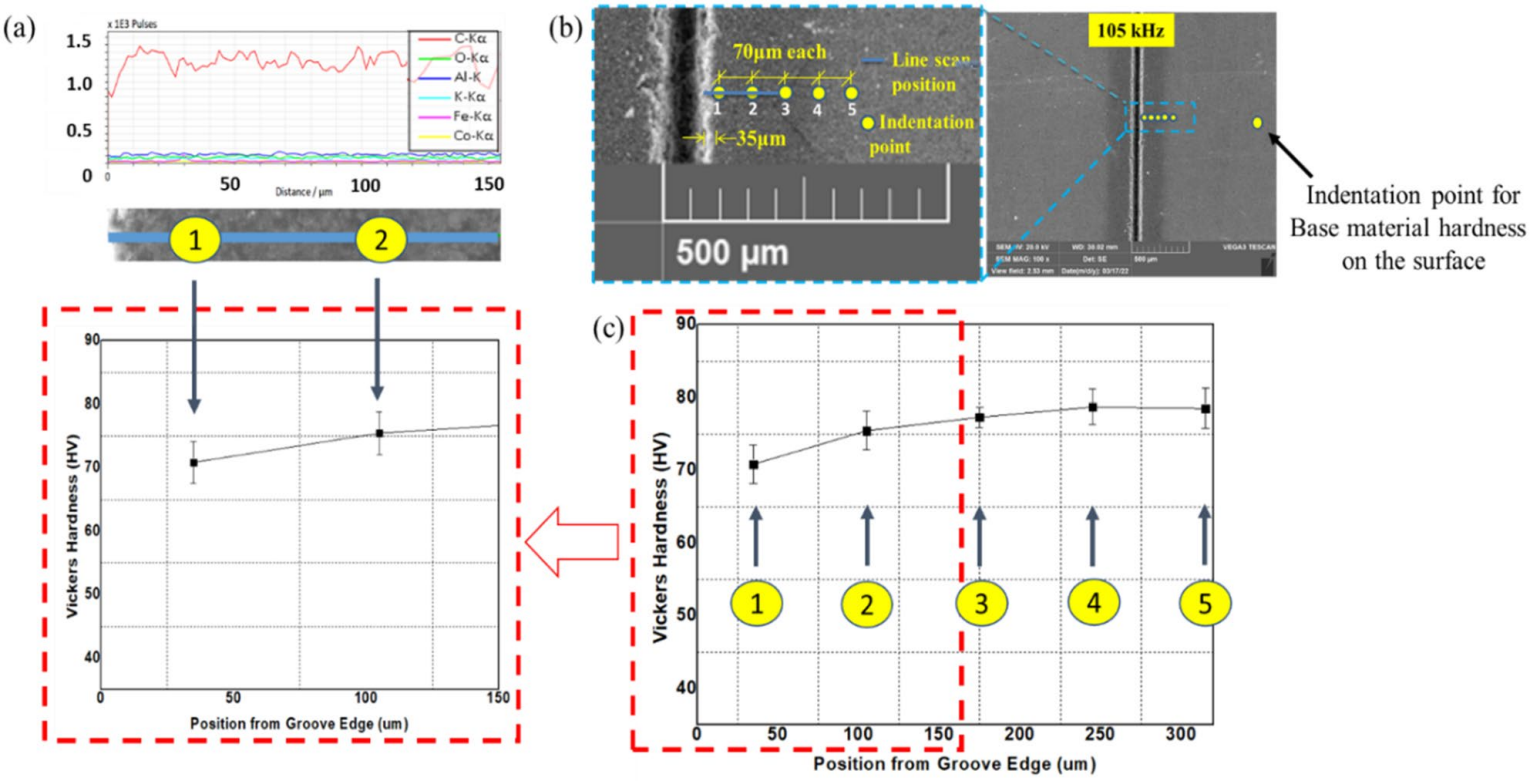
Chemical and hardness tests on surface—105 kHz; (**a**) EDX line scan (**b**) measurement configuration (**c**) Vickers hardness.

**Figure 17:**
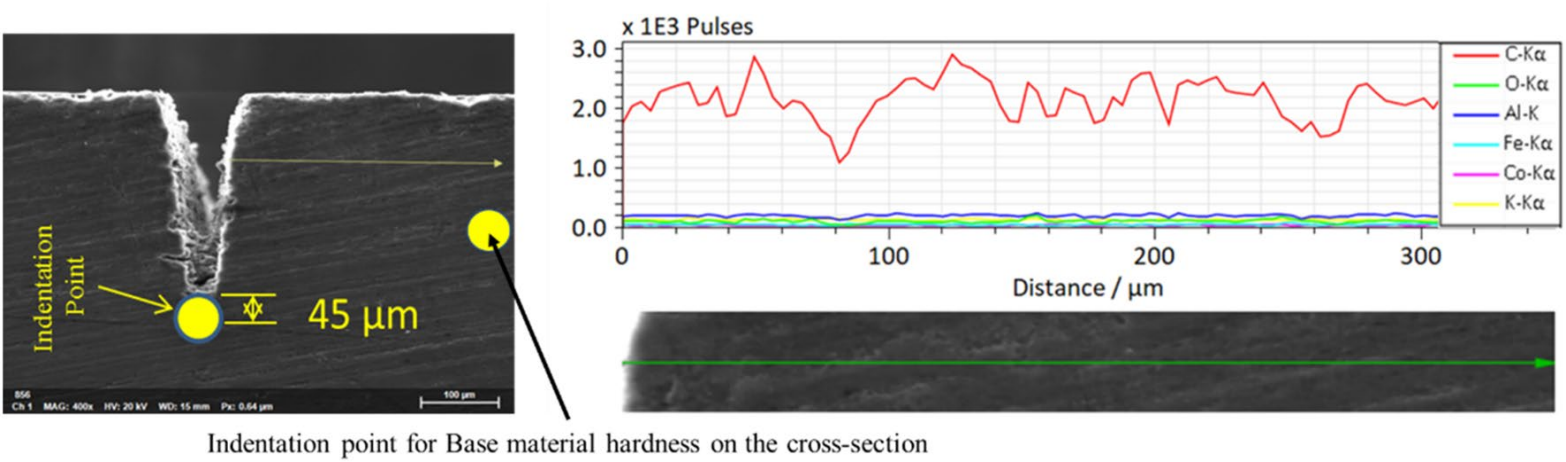
Chemical and hardness tests on cross-section—105 kHz.

This scenario represents the use of repetition rate below 500 kHz. In this case, penetration depth keeps on increasing with pulse repetition rate, and the process does not result in significant thermal effects. However, thermal effects and changes in the material properties can still occur. For this reason, it is essential to investigate the occurrence of a heat-affected zone (HAZ). HAZ is a region created on a material exposed to elevated temperatures. It is a non-melted zone near the exact work area (a region between the melted material and base material) where microstructural changes occur^35^. In the operational regime 1, no evidence of surface melting is noticed, reducing the probability of encountering HAZ. To ensure, EDX line scan mode is performed on the surface and cross-section. On the same line, a microhardness test is conducted using Vickers Microhardness Tester. The experiments will give the chemical composition and mechanical property of the specimen in the region where heat could have an impact. The EDX line scan result of the 105 kHz case shown in Fig. 16a indicates that carbon is the dominant element making other elements miscellaneous. The distribution of carbon over a horizontal line of 150 μm from the edge does not result in a significant change in its content but minor random fluctuations. Also, the difference in other miscellaneous elements is null. On the same line, results of Vickers microhardness are presented in Fig. 16c. These local values are compared to the microhardness of unprocessed base material to determine if the thermal effect is causing hardening, softening, or neither. Since the heat from the laser diffuses laterally on the surface and penetrate down the crossection, it is important to observe the surface and the crossection simultaneously. Multiple vickers hardness measurements are conducted at a fixed intervals from the edge of the groove in a configuration given at Fig. 16b. The hardness tests indicated in Fig. 16c show that hardness values decrease as measured from point 3 to point 1; however, the hardness remains unaffected outside this range (point 3–5). The base material hardness was measured at a random point in the unprocessed region, sufficiently far from point 5 (shown in on Fig. 16b). Accordingly, the Vickers microhardness associated with the base material is determined to be $79.56 \pm 4.2$ HV. It can be noticed that a change in chemical nature and hardness values is taking place. Nearly similar results were found in its cross-section (shown in Fig. 17), where the hardness is found to be 53.1 $\pm 1.2$ HV. The texture and surface condition of the base material on the cross-section is totally different from the top surface. Consequently, the hardness of unprocessed base material in the cross-sectional face is measured to be 52.57 ± 0.92 HV.

**Figure 18:**
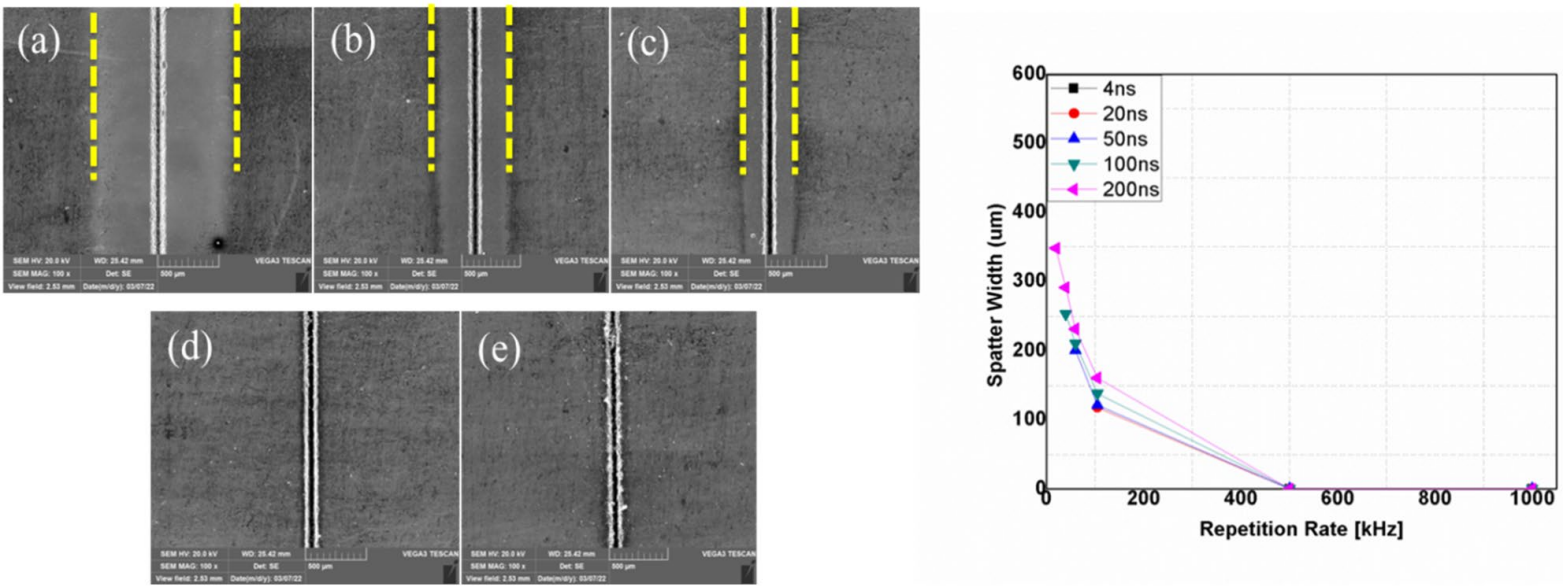
Spatter distribution with repetition rate at 200 ns. (**a**) 20 kHz, (**b**) 60 kHz, (**c**) 105 kHz, (**d**) 500 kHz and (**e**) 1000 kHz [fixed: 20W, 100 mm/s, 1 pass and 200 ns].

**Figure 19:**
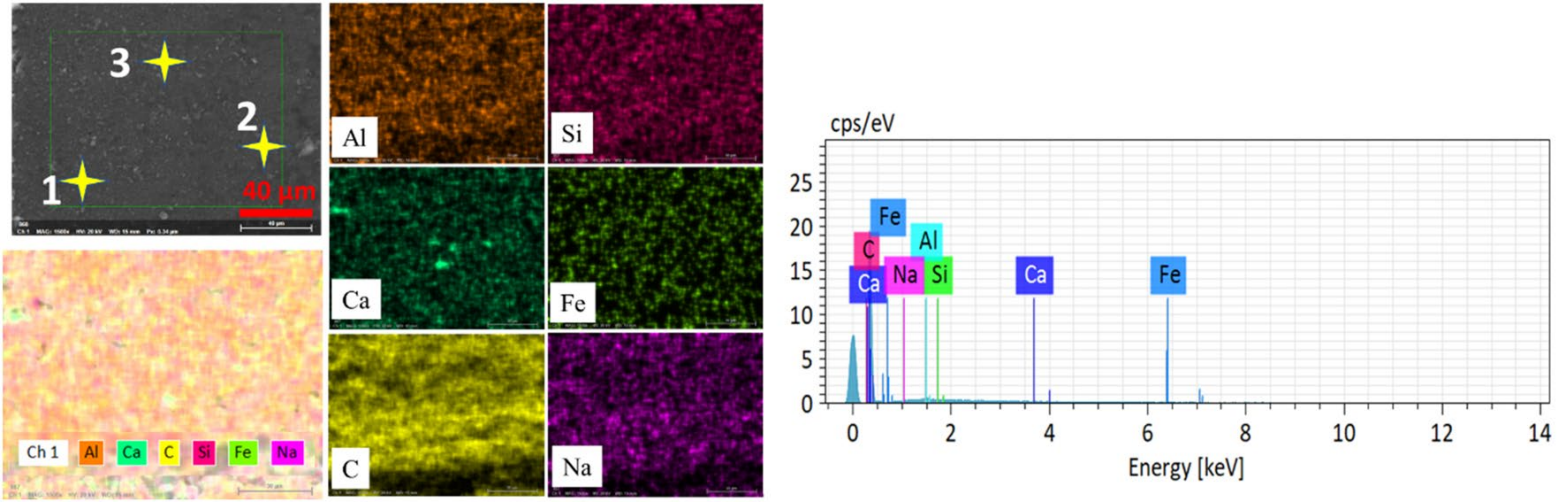
EDX mapping of the spatter.

The carbon concentration on the cross-section and the top surface is different, giving less carbon on the surface. This condition is associated with the formation of a spatter in the regime one. Spatter formation starts in large quantities (higher spatter width) at 20 kHz, decreases in the spatter width up to 105 kHz, and eventually, spatter formation stops at a repetition rate of 500 kHz. The operation from 500 to 1000 kHz is spatter-free. These whole phenomena are indicated in Fig. 18a–e. The EDX elemental mapping and point scan quantifications (averaged) are presented in Fig. 19 and Table 4, respectively. Accordingly, the spatter consists of nearly similar elements with base material but essentially with lower carbon content. In this context, a spatter is a splash of burned and ablated particles from the base CNT composite with small carbon content due to irradiation by high laser pulse energy. Low laser pulses of 500 kHz and above are not observed to yield spatter. Therefore, the underprediction of carbon concentration in the top surface line scan of the 105 kHz case is obviously from the spatter that covers the region.

**Table 4:** Elemental composition of the spatter.

Element	Composition (Weight%)
C	65.11
Al	10.463
Si	12.95
Fe	9.157
Miscellaneous (Na,Ca)	2.32

All these results are related to the nature of pulse repetition. In the pulsed nanosecond laser used for the experiments, repetition rate values of 20–105 kHz can be considered low pulse repetition rate values. During low pulse repetition rate settings, the interval between consecutive pulses is relatively long and gives enough time for the dissipation of heat in the material causing the minimum thermal damage. Also, this allows enough time between pulses for the steady removal of ablated particles. For this reason, a significant amount of spatter is observed outside. However, when the repetition rate increases, the time interval between pulses decreases, and the effectiveness of removing ablated particles decreases. Therefore, the spatter amount keeps dropping to 105 kHz and will eventually disappear after that. Due to this, operations involving repetition rate settings below 500 kHz result in proper heat dissipation and effective conveying of ablated particles to the outside so that the changes in the chemical and mechanical (hardness) nature of the work are minimum.

(ii) Operational regime 2—Operation at a critical pulse repetition rate

**Figure 20:**
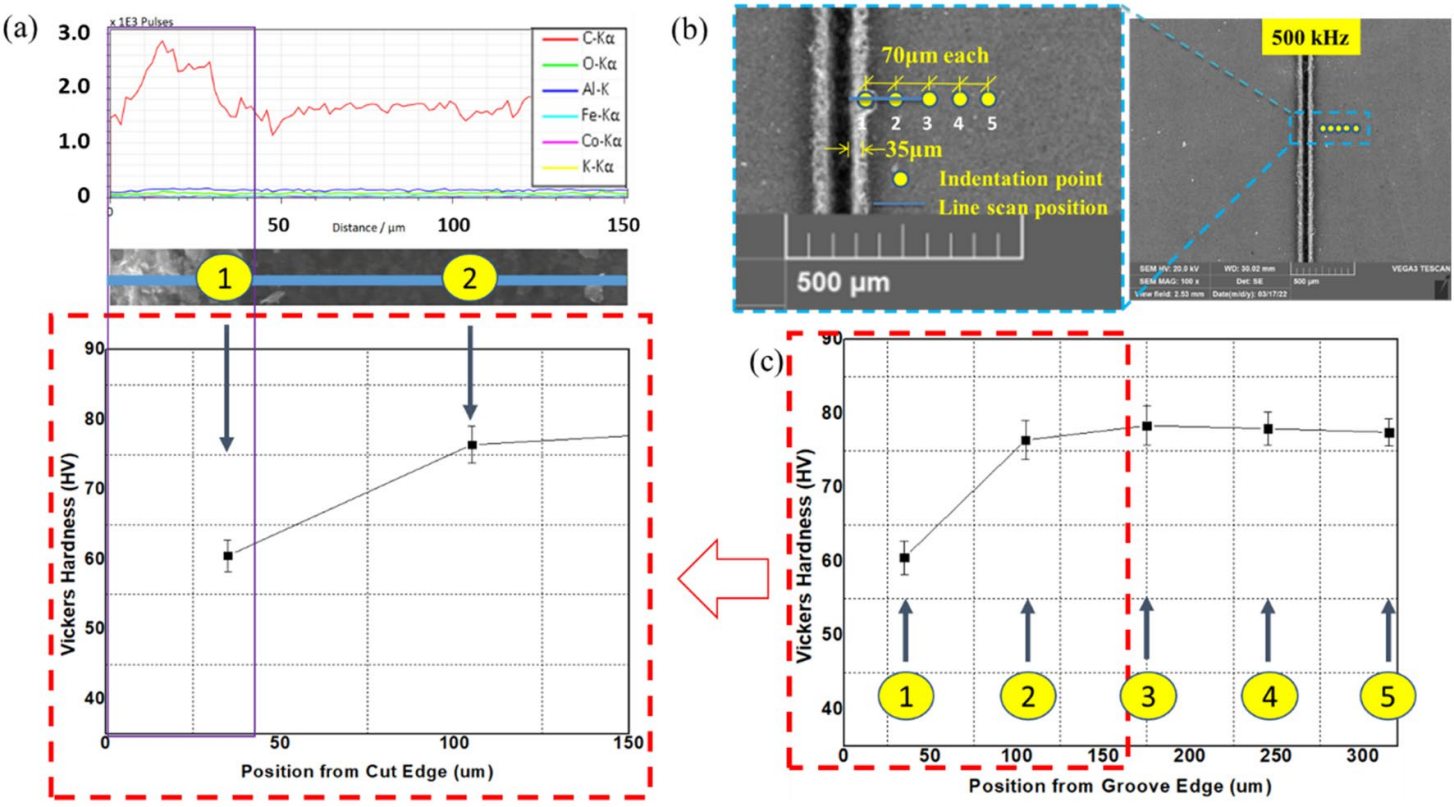
Chemical and hardness tests on surface—500 kHz; (**a**) EDX line scan (**b**) measurement configuration (**c**) Vickers hardness.

**Figure 21:**
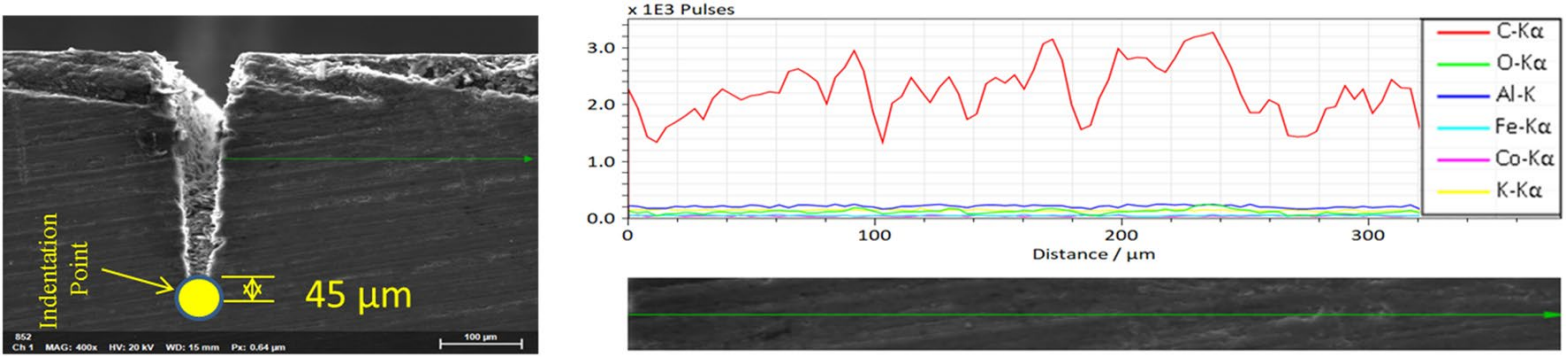
Chemical and hardness tests on cross-section—500 kHz.

Operational regime two depicts operation at a particular point of 500 kHz where the penetration depth attains its maximum value. At this point, thermal effects start to appear along the edge. The evidence of the thermally damaged region observed in Fig. 15d implies heat accumulation due to the increased number of pulses per unit time. On the other hand, the same figure shows no evidence of trapped ablated particles inside the groove as a result of the increased repetition rate. Important features of this region are obtained by inspection through EDX line scan and microhardness testing according to a configuration given in Fig. 20b. Figures 20a–c and 21 show the tests with the results.

Carbon content is the most dominant species in the processed and unprocessed region, but carbon content is observed to be higher in the melted region as quantified and presented in Fig. 20a, thereby giving a more softened surface than the previous scenario (shown on Fig. 20c). Looking at the cross-section, the hardness value in the region was measured to be 44.6 $\pm 1.606$ HV. Comparing this result to the hardness of the base material in the cross-section, $52.57 \pm 0.92$ HV, it can be noted that softening is going on. Operating at regime two gives the maximum ablation depth. This implies the increase in pulse repetition rate is causing thermal effects from heat accumulation resulting from closely spaced pulses. Still, the thermal effect is not observed to hinder the increasing trend of penetration depth. This situation marks the regime as an operational regime to the onset of thermal effects. Also, this regime gives rise to the termination of spatter formation. For these reasons, 500 kHz is marked as a critical repetition rate.

(iii) Operational regime 3—Operation above the critical pulse repetition rate

**Figure 22:**
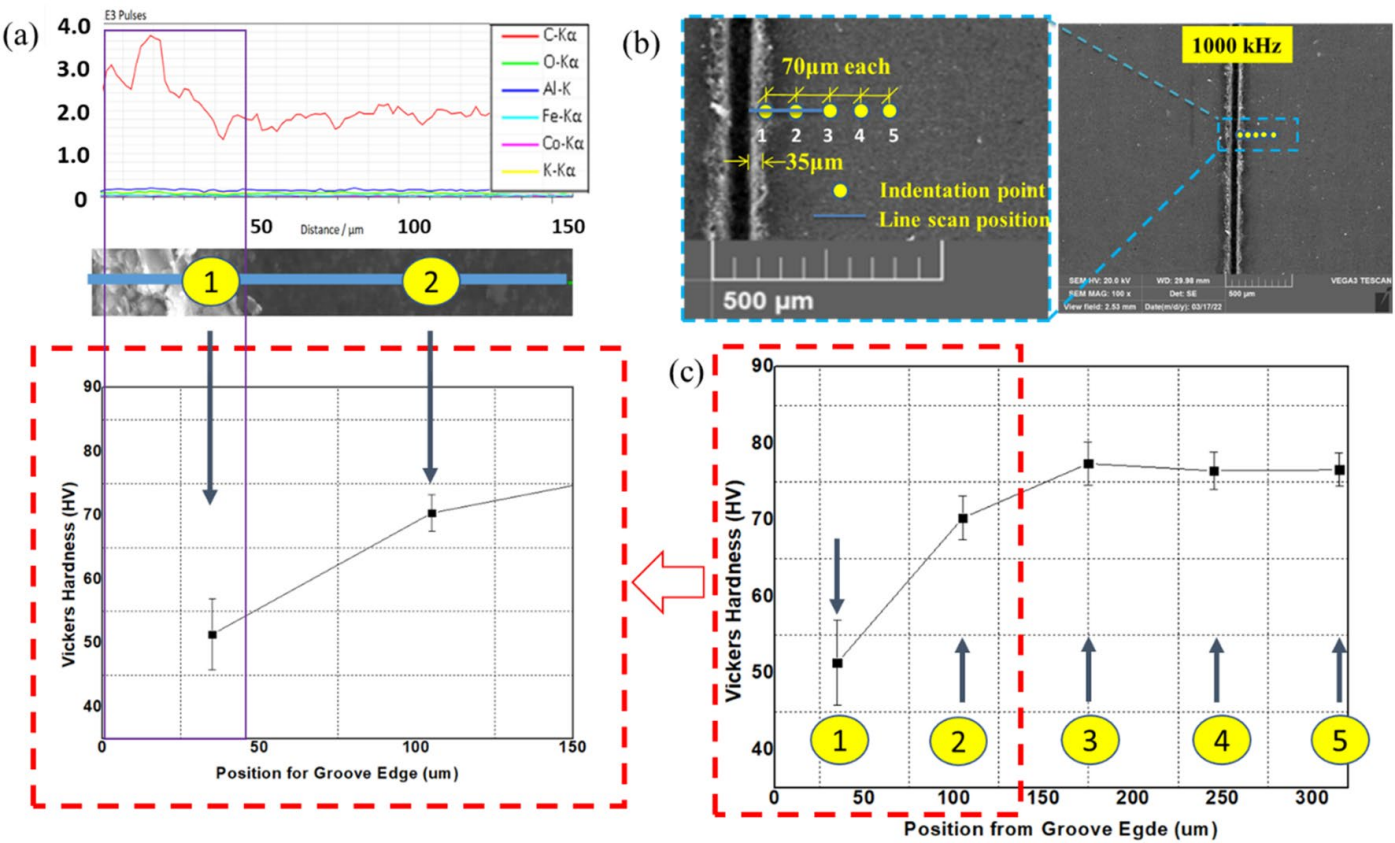
Chemical and hardness tests on surface—1000 kHz; (**a**) EDX line scan (**b**) measurement configuration (**c**) Vickers hardness.

**Figure 23:**
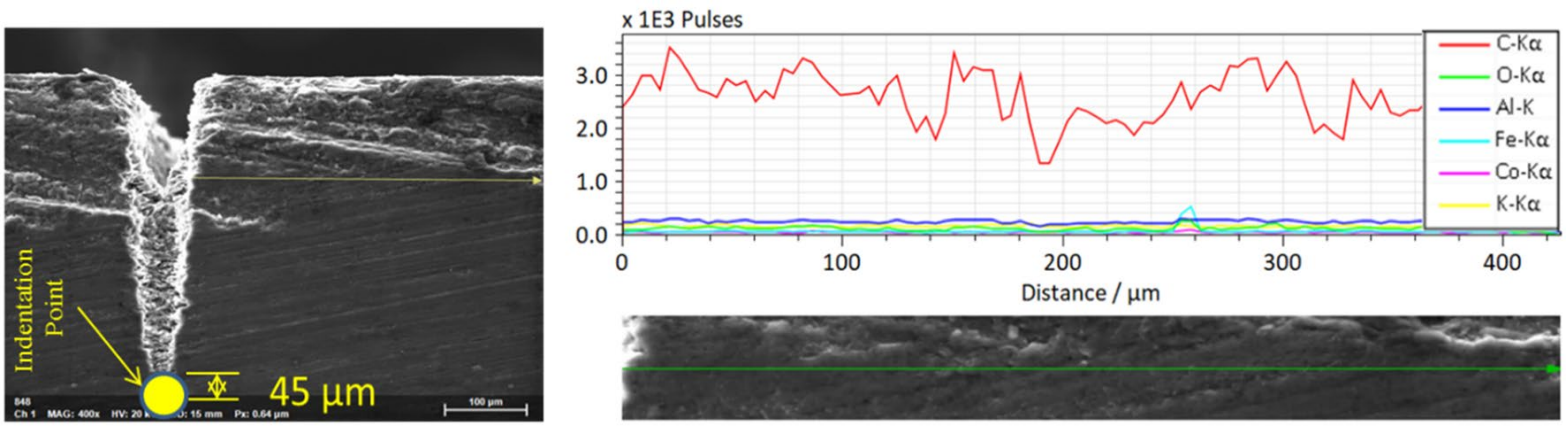
Chemical and hardness tests on cross-section—1000 kHz.

Unlike regimes one and two, in this operational regime (500–1000 kHz], the penetration depth drops. Also, the regime depicts the presence of special characteristics in its morphology at 1000 kHz. Close observation of Fig. 15e reveals that the thermal effect deforms the edges of the groove. Melting of the surface layer near the processed zone has occurred with a subsequent recasting of the layer in the vicinity of the melting region. Also, larger ablated particles inside the groove are visible closely from the top. Following the same procedure, EDX line scan quantifications of the elemental compositions are carried out on the surface and the cross-section, followed by microhardness testing, as shown in Figs. 22a, b, c and 23.

Like the previous case, the thermally damaged region is mainly composed of Carbon; however, the carbon content in this particular region of melting and recast is the highest. Also, the operation has deteriorated the surface hardness of the material in the same region, where it is highly softened. On the other hand, the same observations on the crossectional side yield no change in the chemical composition, but the softening effect is further penetrated inside as it was measured at 38.5 $\pm 3.9$ HV near the processed region compared to $52.57 \pm 0.92$ HV (the base material hardness in the cross-sectional side). As a result, melting with a subsistent formation of recast accompanies the ablation process at the third operational regime. The improper heat accumulation process significantly alters the region's chemical and mechanical nature, resulting in a softened edge with more carbon content.

**Figure 24:**
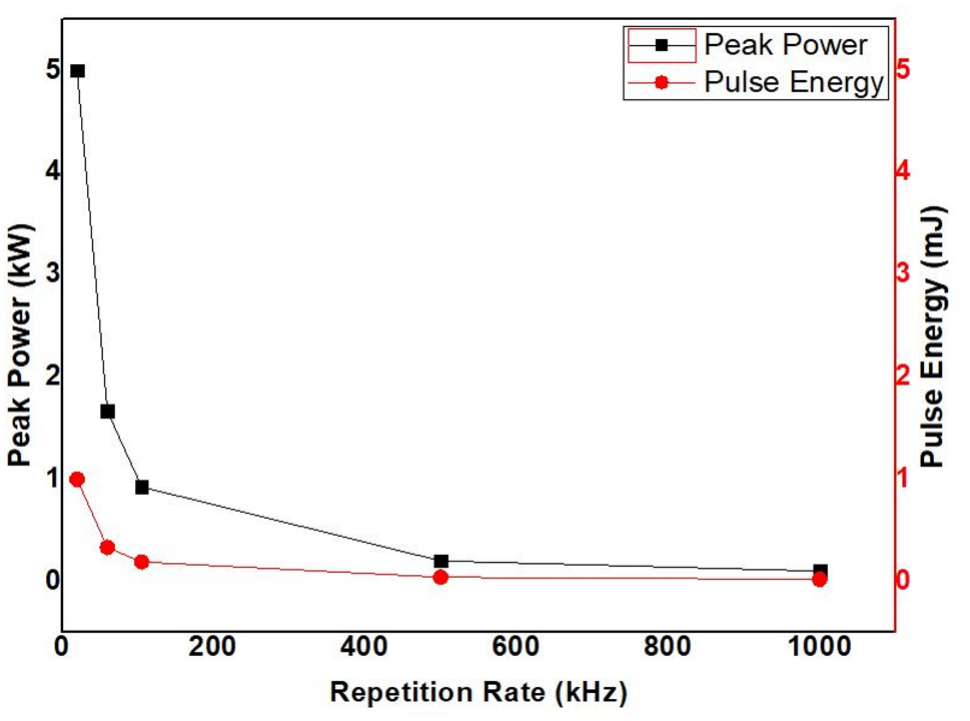
Pulse Energy and Peak Power versus Repetition Rate.

Repetition rate is a measure of the number of pulses emitted per second of a regular train of pulses^36^. The repetition rate is an essential parameter that defines heat utilization for the laser processing of certain materials^37^. Operating at high repetition rate values has its advantages; for instance, in micro-hole drilling using an ultra-pulse laser, higher efficiencies are expected at higher repetition rate since more pulses are radiated at a fixed time^38^. However, excessive repetition rate values cause adverse effects on materials' processability, and the relative magnitudes depend on the material type^37^. In laser processing with high repetition rate values (> 500 kHz), the duration between individual pulses is very short in such a way that thermal energy cannot be adequately dissipated from the heated volume before the arrival of the next pulse^39^. Apart from pulse repetition rate, previous studies indicated that pulse energy and peak power are essential parameters that control the depth and width of a microgroove^34,35^. The pulse energy and peak power values for each repetition rate of 20–1000 kHz are calculated based on Eqs. (1) and (2). The results are plotted and presented in Fig. 24.

1 $$Pulse\;Energy = \frac{{P_{avg} }}{f}$$

2 $$Peak\;Power = \frac{{P_{avg} }}{f \times \tau }$$

where P_avg_, *f*, and $\tau$ are average power, repetation rate, and pulse duration, respectively.

Comparing the results of Fig. 24 with the results of Fig. 13a and b, it is quite evident that the effects of pulse energy and peak power are highly associated with top width. High peak power and pulse energy give larger top width and vice versa. In addition, from 20 to 105 kHz, the top width decreases to a great extent. This is from the sharp reduction of pulse energy and peak power for the same range of repetition rate values. However, the penetration depth increases while the pulse energy decreases from 1–0.04 mJ, and the penetration depth decreases while pulse energy decreases from 0.04 to 0.02 mJ. Also, a similar trend is observed with peak power. This situation is attributed to the two dominant effects of a high pulse repetition rate ^40–43^. The effects are associated with the shortening of the time interval between two successive pulses. This causes: (i) less time for heat diffusion in the material, which introduces a heat accumulation problem in the processed region. Accumulation of excessive heat in the material will not have a contribution to the material removal process but to the formation of morphological changes through the formation of molten spatters and melted regions near the periphery of the processed zone with the subsequent formation of the recast. (ii) less time to remove ablated particles from the processed zone. When the ablated particles are trapped inside the groove due to the shorter time between pulses for effective removal, the upcoming pulses will get shielded. This leads to the melting of these particles. Later, the resolidification of these particles will affect the penetration depth.

Generally, the characteristics observed in the interaction of the laser with the CNT composite can be mapped from the standpoint of laser peak intensity or peak pulse intensity. Laser peak intensity is defined as the maximum power the incident beam delivers per unit area^44^. Laser peak intensity is another crucial working parameter determining ablation rates and other physical phenomena associated with the process^45^. The laser peak intensity is calculated for a single pulse based on Eq. (3). Since laser peak intensity is a function of average power (${P}_{avg}$), pulse duration ($\tau$), and pulse repetition rate ($f$), it could give a more generalized indications on the interaction.

3 $$Laser\;Peak\;Intensity = \frac{{P_{avg} }}{{f \times \tau \times A_{sp} }}$$

where A_sp_ is the laser spot area [mm^2^].

**Figure 25:**
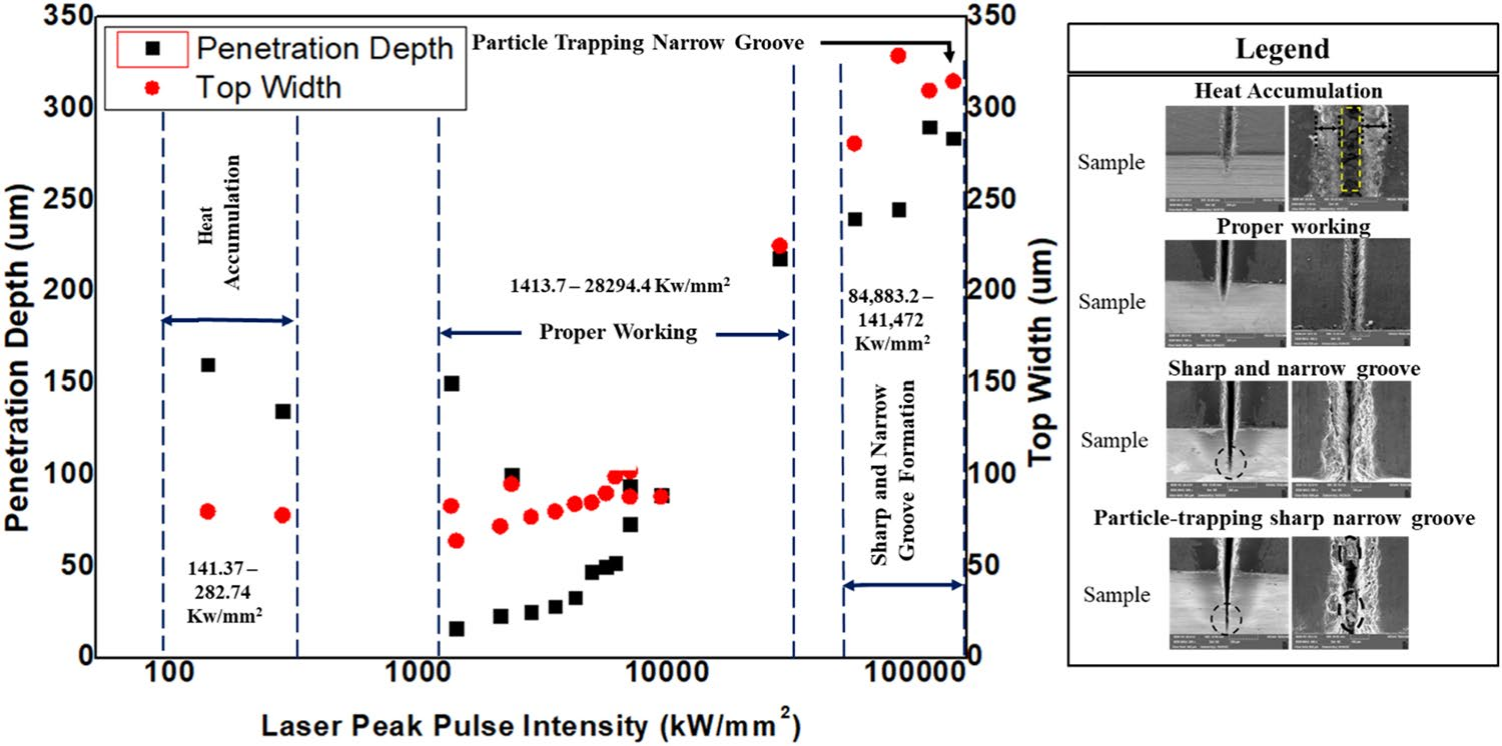
Mapping of laser interaction according of peak intensity.

Figure 25 shows the penetration depth and top width versus peak pulse intensity for the parameters studied from 50 to 200 ns. According to the results, by varying power from 4 to 20W at 20 kHz, peak intensity would be varied from 1413.7 to 7073.6.409 kW/mm^2^ to yield more penetration without causing any additional significant morphological changes. Figure 25 illustrates key features identified by the effect of various laser parameters. Accordingly, heat accumulation regimes discussed earlier cover a peak intensity range of 141.37–282.74 kW/mm^2^. Any parametric combination at a low pulse repetition rate and low number of passes is traced in the range of peak intensity range 1413.7–28,294.4 kW/mm^2^ and identified as a proper working regime. However, the intense peak intensities resulting from the higher number of irradiation due to increasing the number of passes (after 12 passes) results in degradation of the morphological appearance. A range of extremely high peak intensity of 845,883.2–141,472 kW/mm^2^ denotes these operations, suggesting that these high peak intensities introduce undesired effects to the material. Generally, peak intensities ranging from 1413.7 to 28,294.4 kW/mm^2^ can be favored for effective and good-quality operations.

**Figure 26:**
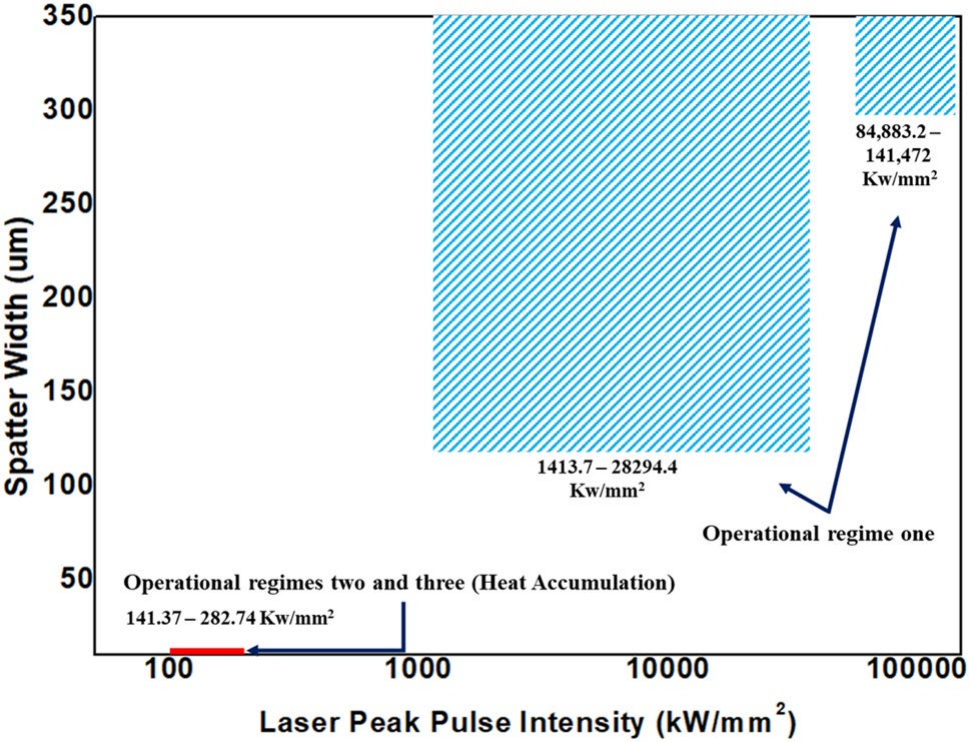
Mapping of laser interaction according of peak intensity and spatter.

Similarly, the laser peak pulse intensities are mapped with the laser parametric combinations to see the effect of laser peak pulse intensity on the top width. It is interesting that the same pulse intensity range (1413.7–28,294.4 kW/mm^2^) provides the proper working regime in this case as well. In the case of the spatter (Fig. 26), it happens while working at 1413.7–141472 kW/mm^2^ peak intensity range on any operation that lies in operational regime one. Operational regimes two and three are already known to give spatter-free operation and cover a very narrow peak intensity range of 141.37–282.74 kW/mm^2^.

In the laser processing of polymeric composites, intense laser beams tend to break the polymer chains and introduce process defects and failures in the material^46^. Incorporating CNTs in composites plays a vital role in their processability by increasing the hardness and laser absorptivity of composites^47^. It has been reported that laser processing of carbon-based composites suffers from thermal effects due to large differences in the thermal conductivity and vaporization temperature between the carbon and the matrix^48^. Besides, the latent heat capacity of carbon is 43 times that of the epoxy resin, implying that carbon will absorb most of the heat^49^. The absorbed thermal energy will then be conducted along carbon fibers, and the epoxy resin will evaporate by the heat even in the non-processed region. This situation marks the formation of HAZ, and material removal became complicated^48^. This study demonstrated that significantly high laser beam intensities could be employed for proper material removal (Fig. 25), which emphasizes the role of CNTs in absorbing part of the thermal energy input and providing additional pathways^50^ for effective heat conduction. This mechanism is believed to significantly reduce the thermal effects on laser processability.

## Conclusion

This study has presented an experimental parametric study on laser interaction with CNT composite plate for use as bipolar plate in PEMFCs by considering a 2.5 mm thick plate. The basic concluding remarks outlined by the study are:

- The effect from the number of passes is the highest in controlling penetration depth and top width. Additionally, care must be taken to render a high value of the number of passes as it is indicated to be a negative factor for quality operation.
- Relatively smooth operations resulting from power and scanning speed indicate that these parameters are adequate for low to medium control of penetration depth and top width without significant impact on morphological aspects.
- Pulse repetition rate presents a unique nature of interaction that resulted in a critical repetition rate distinguishing three different regimes: below the critical value, at the critical value, and above the critical value, each with certain distinct chemical and mechanical behaviors.
- Operations that favored repetition rate values below the critical value yielded reliable results.

Typical channel dimensions of flow channels on bipolar plates for the rectangular slot were reported as 300 μm width and 300 μm height^51^. Overall, the ablation characteristics suggest that laser processing can be considered a potential method for manufacturing flow channels of bipolar plates using CNT composite materials.

## Data Availability

The datasets used and/or analyzed during the current study are available from the corresponding author on reasonable request.
